# Enhanced expression of spinal PTP1B contributes to peripheral inflammation- induced glycinergic disinhibition and mechanical pain

**DOI:** 10.1038/s42003-026-10318-5

**Published:** 2026-05-20

**Authors:** Hu-Hu Bai, Xue Bai, Xiao-Xue Liu, Yu-Bo Gao, Juan Li, Xu Yang, Jia-Ning Dang, Ying Wang, Yu-Xuan Zhang, Zhi-Yang Zhang, Qi Zhang, Yan-Ni Liu, Jiang-Ping Liu, Zhan-Wei Suo

**Affiliations:** 1 Department of Molecular Pharmacology, School of Pharmacy, Lanzhou University, Lanzhou, Gansu PR China; 2 NHC Key Laboratory of Diagnosis and Therapy of Gastrointestinal Tumor, Institute of Clinical Research and Translational Medicine, Gansu Provincial Hospital, Lanzhou, Gansu China

**Keywords:** Sensory processing, Phosphorylation

## Abstract

Spinal glycinergic disinhibition represents an important contributor to mechanical pain hypersensitivity. However, the molecular mechanisms through which peripheral inflammation induces glycinergic disinhibition remain incompletely understood. Here we show that the protein tyrosine phosphatase-1B (PTP1B) is present at inhibitory synapses of spinal somatostatin-positive (SOM^+^) interneurons, a key subpopulation of mechanosensory neurons that conveys nociceptive information. PTP1B directly binds to and dephosphorylates the glycine receptor α1 subunit (GlyR α1). Peripheral inflammation-induced hyperexpression of PTP1B competitively interrupts the interaction of GlyR α1 with GPR39, leading to glycinergic disinhibition, enhanced excitatory output of SOM^+^ interneurons, and behavioral hypersensitivity. Pharmacological activation of GPR39, however, could disrupt the PTP1B-GlyR α1 interaction and resume GPR39 binding, resulting in reinstated glycinergic inhibition. Our findings uncover the important role of PTP1B in glycinergic disinhibition following peripheral inflammation and suggest a mechanistic model for dynamic regulation of glycinergic transmission by alternative interactions of GlyR α1 with PTP1B and GPR39.

## Abstract (web summary)

Alternative interactions of GlyR α1 with PTP1B and GPR39 in somatostatin-positive interneurons of spinal dorsal horn reveal how peripheral inflammation leads to glycinergic disinhibition and mechanical pain hypersensitivity

## Introduction

Peripheral inflammation and nerve injury result in spinal sensitization and nociceptive hypersensitivity^1^. One typical sensory symptom is mechanical pain hypersensitivity that manifests as an increased sensitivity to innocuous mechanical stimuli or movement. According to the gate control theory of pain, spinal nociceptive transmission is gated by GABAergic or glycinergic inhibitory interneurons in the dorsal horn^2^. Under physiological conditions, these inhibitory interneurons are activated by the primary afferent low-threshold mechanoreceptors (LTMRs) and act to reduce the pain transmission from mechanosensory interneurons to projection neurons^3^. As a representative population of mechanosensory interneurons, the somatostatin-positive (SOM^+^) excitatory neurons play a crucial role in the conveyance of mechanosensory information^4,5^. Following tissue or nerve injury, spinal GABAergic or glycinergic disinhibition allows LTMR input to activate SOM^+^ interneurons^6–10^. However, the cellular and molecular mechanisms of the glycinergic disinhibition remain incompletely understood.

G protein-coupled receptor 39 (GPR39) belongs to the ghrelin/neurotensin receptor family with a high degree of constitutive activities toward Gαq/11, Gα12/13, and Gαs proteins^11,12^. Accumulating evidence indicates that GPR39 engages multiple G protein signaling pathways to modulate synaptic strength and neuronal excitability, and its dysregulation is implicated in several psychiatric disorders^12–14^. Activation of GPR39 in the spinal cord alleviates neuropathic and chronic inflammatory pain^10,15,16^. A recent study shows that a substantial pool of GPR39 is located in inhibitory synapses of spinal SOM^+^ neurons, where it directly interacts with glycine receptor α1 subunit (GlyR α1) and positively regulates glycinergic transmission in a G protein-independent manner^10^. Pharmacological activation of GPR39 or enhancement of the GPR39-GlyR α1 interaction alleviates mechanical pain behaviors via potentiating glycinergic transmission^10^, but the molecular mechanism remains to be revealed.

Phosphorylation modification is a crucial regulatory mechanism for ion channel function and is involved in diverse biological and pathological processes. GlyR α1 is found to harbor putative phosphorylation residues that are implicated in the activity-dependent modification of glycinergic efficacy^17,18^. The tyrosine phosphorylation at Tyr339 within the intracellular large loop of GlyR α1 is a key event that required for glycinergic transmission^10^. Multiple protein tyrosine phosphatases (PTPs) have been found in spinal cord dorsal horn^19,20^, but the exact roles of PTPs in regulating glycinergic transmission remain to be elucidated. Here, we identified a specific interaction of protein tyrosine phosphatases-1B (PTP1B) with GlyRs, and revealed a mechanistic pattern for the dynamic regulation of glycinergic transmission. Our data showed that PTP1B and GPR39 competitively interacted with GlyR α1 to regulate the tyrosine phosphorylation of GlyR α1 and glycinergic inhibition. Pharmacological activation of GPR39 reversed glycinergic disinhibition and alleviated mechanical pain via disrupting the PTP1B-GlyR α1 interaction in complete Freund’s adjuvant (CFA)-induced inflammation pain and spared nerve injury (SNI)-induced neuropathic pain model.

## Results

### PTP1B interacted with GlyR α1 in SOM^+^ interneurons of the spinal cord dorsal horn

**Fig. 1:**
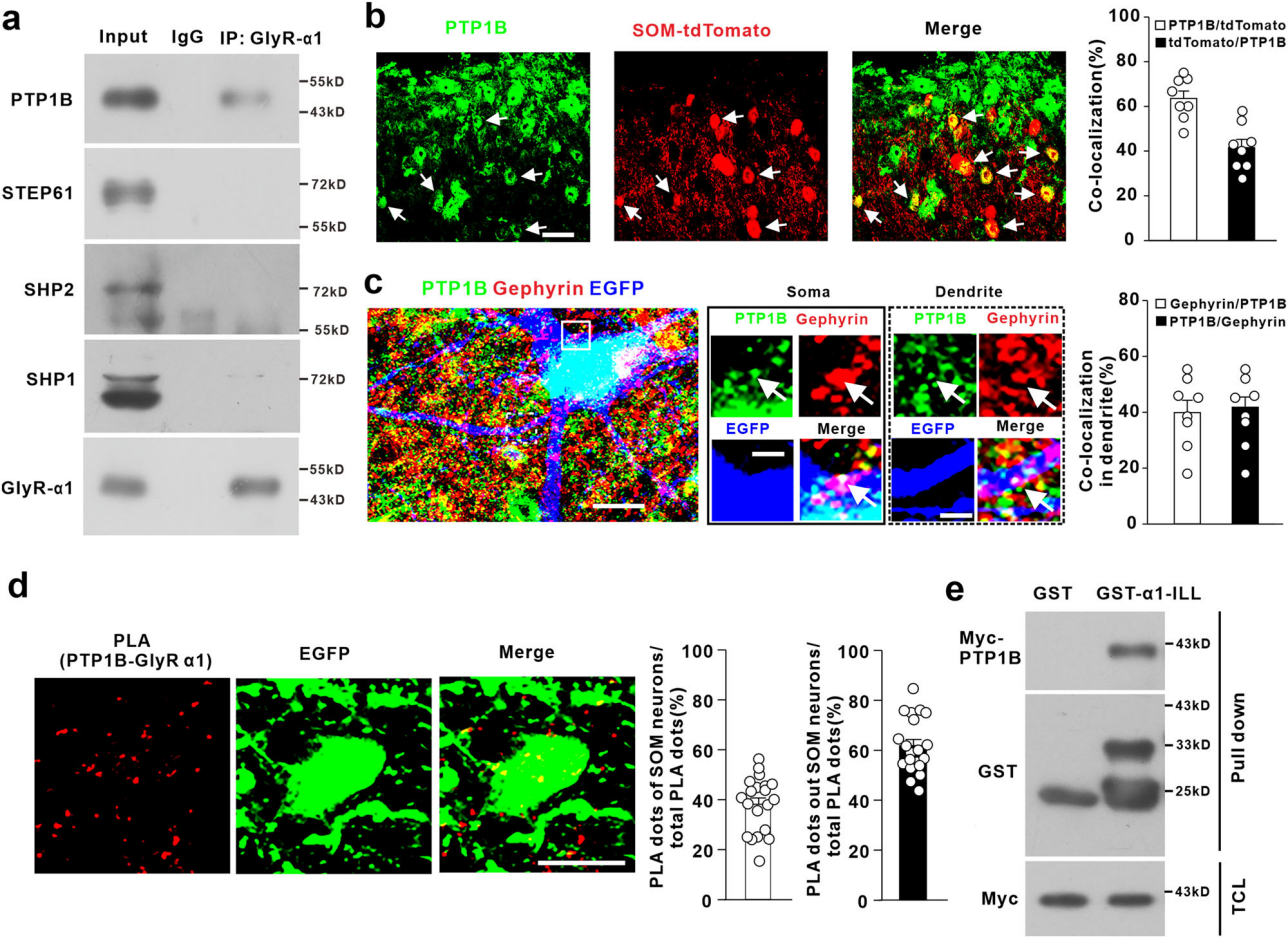
PTP1B interacted with GlyR α1 in the spinal cord dorsal horn of mice. **a** Co-immunoprecipitation (Co-IP) with a specific antibody against GlyR α1 from the lysates of the spinal dorsal horn, and the immunoprecipitates were immunoblotted. The experiments were replicated three times, and each obtained a similar result. IP: immunoprecipitation. **b** Immunostaining of PTP1B (green) in spinal SOM^+^ neurons (red) from SOM-tdtomato mice. Arrows indicated the colocalization. The graph summarizes the percentage of tdTomato^+^ neurons expressing PTP1B (PTP1B/tdTomato) or the percentage of PTP1B ^+^ neurons co-localizing with tdTomato (tdTomato/PTP1B). *n* = 8 sections from three mice. Scale bar, 20 μm. **c** Immunofluorescence for PTP1B (green) and gephyrin (red) in SOM^+^ neurons (blue) at days 21 after intraspinal injection of AAV2/9- EF1α-DIO-EGFP in SOM-Cre mice. The boxed area (left) was shown with higher magnification (middle). The arrow indicated the colocalization. The graph summarizes the percentage of PTP1B signals expressing gephyrin (gephyrin/PTP1B) or the percentage of gephyrin signals co-localizing with PTP1B (PTP1B/gephyrin). *n* = 8 sections from three mice. Scale bars, 10 μm (left) and 2 μm (middle). **d** PLA detection of PTP1B-GlyR α1 complex in EGFP-labeled SOM^+^ interneurons. Scale bar, 10 µm. **e** GST-fused intracellular large loop of GlyR α1 (GST-α1-ILL) pulled down Myc-PTP1B(1-321/D181A) from transfected HEK293T cells. The experiments were replicated three times, and each obtained a similar result. TCL total cell lysates. All data were shown as mean ± SEM.

A previous study has indicated that GPR39 might maintain glycinergic transmission by blocking protein tyrosine phosphatases (PTPs) from dephosphorylating GlyR α1 in somatostatin-positive (SOM^+^) interneurons^10^. We therefore first identified the PTPs that might interact with GlyR α1. Co-immunoprecipitation showed that the specific antibody of GlyR α1 immunoprecipitated PTP1B from the lysates of the spinal dorsal horn of normal mice (Fig. 1a). By contrast, the same antibody failed to co-immunoprecipitate STEP 61, SHP1 and SHP2 (Fig. 1a). To test whether PTP1B was expressed in SOM^+^ interneurons, the SOM^+^ neurons were labeled with tdTomato by crossing SOM-Cre mice with Ai14 reporter mice (SOM-tdTomato mice). Immunofluorescence showed that the majority of SOM^+^ neurons expressed PTP1B (Fig. 1b), and a small portion of PTP1B^+^ neurons were positive for tdTomato. PTP1B was also present at inhibitory postsynapses, as shown in Fig. 1c, the PTP1B immunoreactivity was detected in gephyrin-positive synapses of SOM^+^ interneurons labeled by injecting AAV2/9-EF1α-DIO-EGFP into the lumbar spinal cords of SOM-Cre mice.

To confirm the interaction of PTP1B with GlyR α1 in SOM^+^ interneurons, the proximity ligation assay (PLA) was conducted in EGFP-labeled SOM^+^ neurons from spinal sections of SOM-Cre mice. The punctate PLA signal of PTP1B-GlyR α1 proximity was densely present in SOM^+^ interneurons (Fig. 1d). The intracellular large loop (ILL) is a key structural motif of GlyR α1 subunit, which plays critical roles in the recruitment of intracellular signaling components^21^. We next tested if PTP1B directly interacted with ILL of GlyR α1 (GlyR α1-ILL) by using the glutathione *S*-transferase (GST) pull-down assay in vitro. Our data showed that the GlyR α1-ILL pulled down Myc-tagged PTP1B (Myc-PTP1B) from the lysates of transfected human embryonic kidney (HEK) 293T cells (Fig. 1e), suggesting an interaction of PTP1B with GlyR α1-ILL.

### PTP1B negatively regulated glycinergic transmission by dephosphorylating of GlyR α1 at Tyr339

**Fig. 2:**
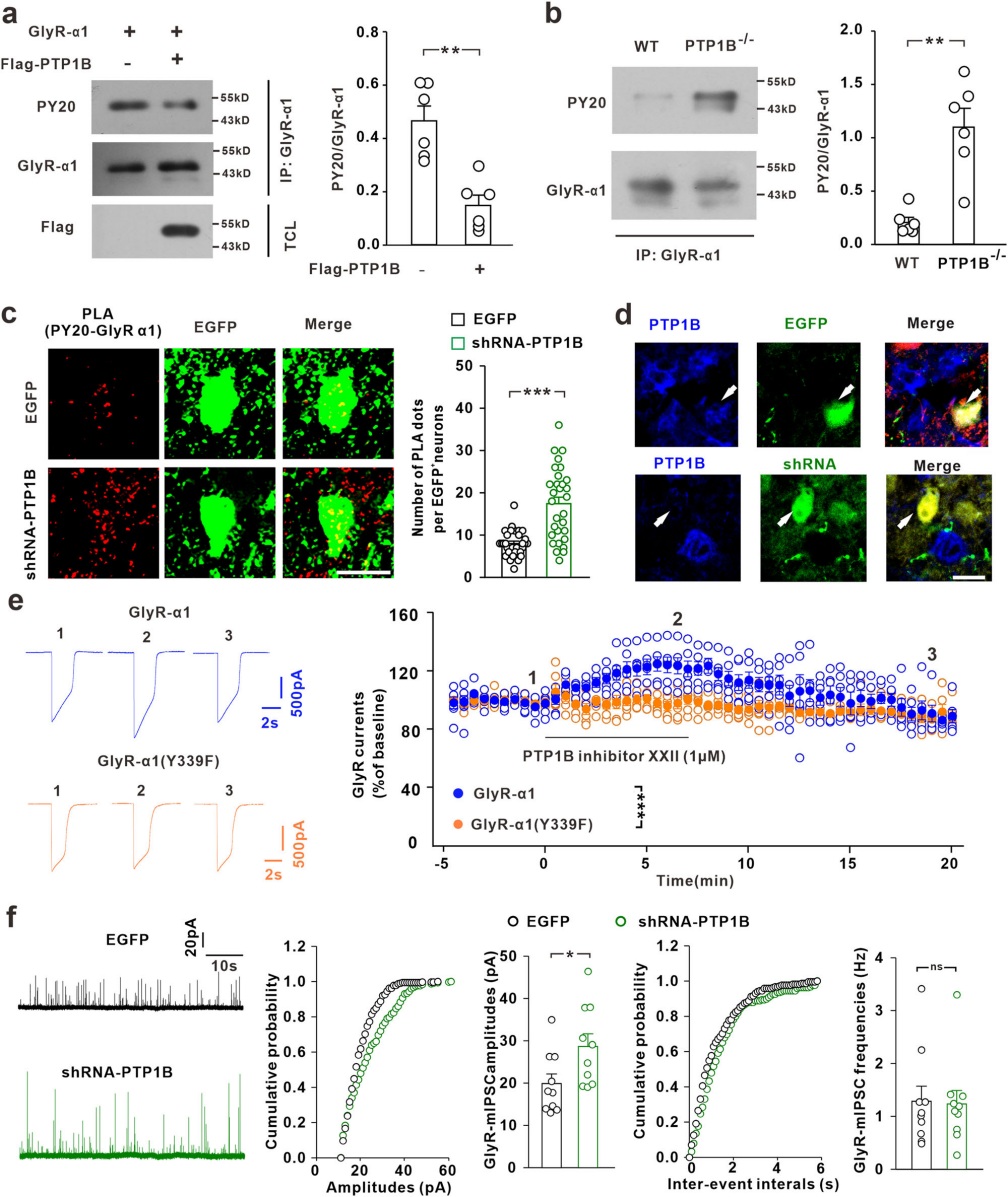
PTP1B negatively regulated glycinergic transmission in spinal SOM^+^ neurons. **a** GlyR α1 immunoprecipitates from HEK293T cells co-expressing Flag-tagged PTP1B (Flag-PTP1B) were immunoblotted for PY20 and GlyR α1. TCL total cell lysates, IP immunoprecipitation. **P = 0.004 (Mann–Whitney *U*-test), *n* = 6 experiments. **b** GlyR α1 immunoprecipitates from spinal dorsal horn of PTP1B^−/−^ and wild-type (WT) mice were immunoblotted for PY20 and GlyR α1. **P = 0.004 (Mann–Whitney *U*-test), *n* = 6 experiments. **c** PLA assay of GlyR α1 tyrosine phosphorylation with a pair of PY20 and GlyR α1 antibody in spinal SOM^+^ interneurons transfected with EGFP or shRNA-PTP1B. ***P < 0.001 (Mann–Whitney *U*-test). *n* = 30 neurons from three mice per group. Scale bar, 10 μm. **d** Spinal sections showing PTP1B (blue) and EGFP at days 21 after intraspinal injection of AAV2/9-EF1α-DIO-EGFP (EGFP) or AAV2/9-EF1α-DIO-shRNA-PTP1B-EGFP (shRNA-PTP1B) from SOM-Cre mice. Arrows indicated the transfected SOM^+^ neurons. Scale bar: 10 µm. **e** Effects of extracellular perfusion of PTP1B inhibitor XXII (horizontal bar) on glycinergic currents mediated by GlyR α1 and GlyR α1(Y339F) in HEK293T cells. The original traces were taken at the time points indicated by the numbers 1–3. F_(49,490)_ = 3.323, ***P < 0.001 (Repeated measurement). *n* = 6 cells per group. **f** GlyR-mIPSCs were recorded on SOM^+^ neurons expressing EGFP or shRNA-PTP1B in SOM-tdTomato mice. *P = 0.023, ^ns^*P* = 0.853 (Mann–Whitney *U*-test), *n* = 10 neurons from six mice per group. All data were shown as mean ± SEM.

The direct interaction of PTP1B with GlyR α1 prompted us to test if PTP1B catalyzed the dephosphorylation of GlyR α1. Since the tyrosine residue Tyr339 within the intracellular large loop of GlyR α1 is the unique site for tyrosine phosphorylation, the detection of phosphorylation at this site can be achieved by using anti-phosphotyrosine antibody (PY20)^10^. We examined the tyrosine phosphorylation levels of GlyR α1 in HEK293T cells co-transfected with GlyR α1 and Flag-tagged PTP1B. Compared with the control group, expression of PTP1B significantly decreased the tyrosine phosphorylation of GlyR α1 (Fig. 2a). Consistent with this observation, there was a marked enhancement in GlyR α1 tyrosine phosphorylation in the spinal cord dorsal horn of PTP1B^−/−^ mice relative to the wild-type (WT) controls (Fig. 2b).

To observe the dephosphorylation of GlyR α1 by PTP1B in SOM^+^ interneurons, we performed an in situ PLA assay in spinal sections using a pair of antibodies against GlyR α1 and phosphotyrosine (PY20). The SOM^+^ interneurons were labeled by injecting AAV2/9-EF1α-DIO-EGFP into the lumbar spinal cord of SOM-Cre mice. The PLA signals appeared as discrete puncta within EGFP-positive neurons (Fig. 2c). The PLA (GlyR α1 + PY20) puncta were substantially increased when PTP1B was knocked down by viral delivery of shRNA-PTP1B in SOM^+^ interneurons (Fig. 2c), suggesting that PTP1B could catalyze the tyrosine dephosphorylation of GlyR α1. Immunostaining demonstrated that shRNA-PTP1B effectively reduced the protein levels of PTP1B in SOM^+^ neurons at day 21 after viral injection (Fig. 2d).

Given that PTP1B directly binds to and dephosphorylates GlyR α1, we wanted to know if PTP1B regulated the function of GlyR α1. We co-transfected GlyR α1 and PTP1B into HEK293T cells. Electrophysiological recordings showed that extracellular perfusion of PTP1B inhibitor XXII significantly increased the amplitudes of GlyR α1 currents (Fig. 2e), suggesting a negative control over GlyR α1 transmission by PTP1B. When the tyrosine residue (Tyr339) within the ILL of GlyR α1 was mutated to phenylalanine (Y339F), however, the PTP1B inhibition failed to potentiate GlyR α1 currents (Fig. 2e), indicating that PTP1B regulated GlyR α1 currents by dephosphorylating GlyR α1 at Tyr339. To evaluate the role of PTP1B in glycinergic transmission in SOM^+^ interneurons, PTP1B was knocked down by intraspinal injection of AAV2/9-EF1α-DIO-shRNA-PTP1B-EGFP in SOM-tdTomato mice. The amplitudes of GlyR-mIPSCs in the shRNA-PTP1B group were significantly increased compared to the EGFP group (Fig. 2f), while the frequencies of GlyR-mIPSCs, an indicator of presynaptic release probability, were unaltered, suggesting a postsynaptic origin. In addition, PTP1B knockdown also increased GABAergic transmission in spinal SOM^+^ interneurons (sFig. 1). These data confirmed an intrinsic inhibition of glycinergic transmission by PTP1B.

To test whether PTP1B ablation causes the hyperexcitability of SOM^+^ neurons under physiological conditions, we recorded the spontaneous action potentials (sAPs) of spinal SOM^+^ neurons from naïve SOM-tdTomato mice. No change of the sAPs firing frequency of SOM^+^ neurons was observed following PTP1B knockdown (Suppl Fig. 2), suggesting that PTP1B knockdown under physiological conditions did not alter the basal excitability of SOM^+^ neurons. Presumably, the low basal activity or expression of PTP1B under resting conditions might account for the lack of enhanced excitability in SOM^+^ neurons following PTP1B knockdown in naïve mice.

### Increased expression of PTP1B contributed to the inflammation-induced glycinergic disinhibition

**Fig. 3:**
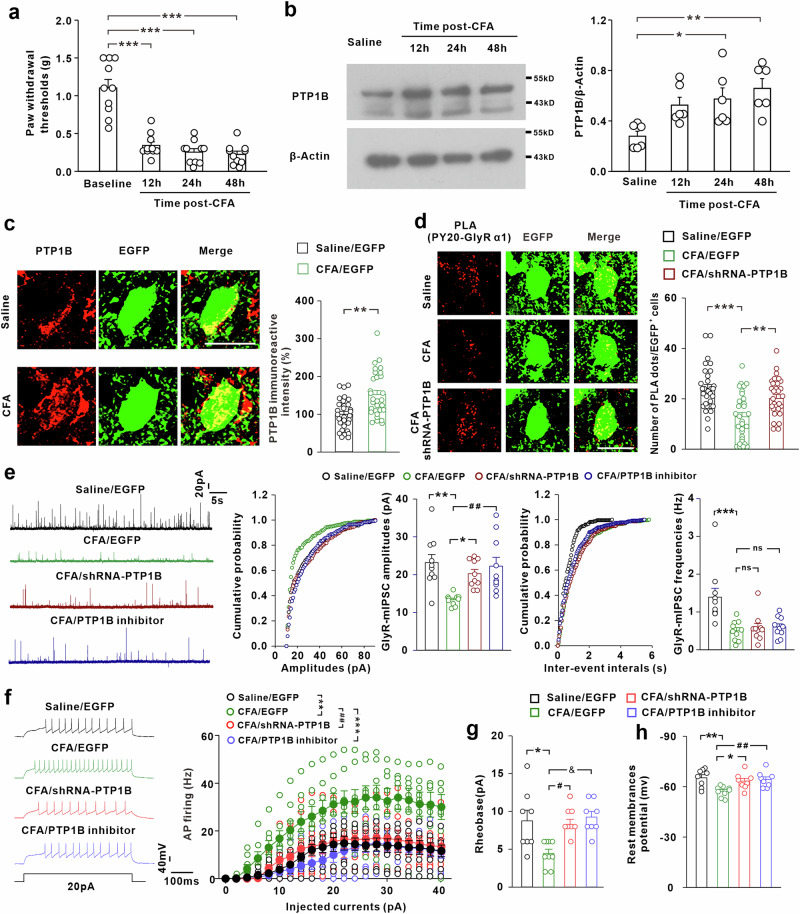
Upregulated expression of PTP1B contributed to the peripheral inflammation-induced glycinergic disinhibition. **a** von Frey thresholds before (baseline) and after Spared Nerve Injury (SNI). ***P < 0.001 (one-way ANOVA and post hoc Bonferroni test). *n* = 10 mice/group. **b** Temporal changes of spinal PTP1B protein levels after CFA injection. *P = 0.042, **P = 0.006 (One-way ANOVA and post hoc Bonferroni test), *n* = 6 experiments. **c** Immunohistochemistry for PTP1B (red) in spinal SOM^+^ neurons (green) at 24 h post CFA injection. ***P = 0.001 (Mann–Whitney *U*-test). *n* = 30 neurons from three mice per group. Scale bar, 10 μm. **d** PLA assay of GlyR α1 tyrosine phosphorylation in SOM^+^ interneurons expressing EGFP or shRNA-PTP1B at 24 h after saline or CFA injection. **P = 0.005, ***P < 0.001 (one-way ANOVA and post hoc Bonferroni test). *n* = 30 neurons from three mice per group. Scale bar, 10 μm. **e** GlyR-mIPSCs record**e**d on SOM^+^ neurons expressing EGFP or shRNA-PTP1B at 24 h after saline or CFA injection in SOM-Cre mice. ***P < 0.001, ^##^*P* = 0.004, **P = 0.001, *P = 0.041, ^ns^*P* = 1.00 (one-way ANOVA and post hoc Bonferroni test), *n* = 10 neurons per group from 3–4 mice. **f** Current-clamp recordings of action potential (AP) firings elicited by depolarizing current injections in SOM^+^ interneurons expressing EGFP or shRNA-PTP1B at 24 h after saline or CFA injection (left). The firing frequencies at all current injection levels were quantified (right). F_(60,560)_ = 2.453, ***P < 0.001 (repeated measurement); **P = 0.001, ^##^*P* = 0.006, ***P < 0.001 (post hoc Bonferroni test). *n* = 8 neurons from 3 mice per group. (**g**, **h**) Rheobase (**g**, *P = 0.01, ^#^*P* = 0.025, ^&^*P* = 0.022) and resting membrane potential ^(^h; *P = 0.031, **P = 0.001, ^##^*P* = 0.004) of SOM^+^ interneurons expressing EGFP or shRNA-PTP1B at 24 hours after saline or CFA injection. One-way ANOVA and post hoc Bonferroni test, *n* = 8–9 neurons from three mice per group. All data were shown as mean ± SEM.

To investigate the role of PTP1B in inflammatory pain, CFA was subcutaneously injected into the plantar surfaces of the hindpaws of mice. The von Frey test showed that CFA caused a time-dependent reduction of the paw withdrawal thresholds (PWTs) (Fig. 3a). Along with the declines in PWTs, the total expression levels of PTP1B in the spinal dorsal horn were increased in a time-dependent manner (Fig. 3b). Moreover, in EGFP-labeled SOM^+^ interneurons of the spinal dorsal horn, a significant increase in PTP1B signals occurred at 24 h after CFA injection (Fig. 3c).

To test if the peripheral injury-induced upregulation of PTP1B influenced the tyrosine phosphorylation of GlyR α1 in SOM^+^ interneurons, EGFP was transfected in SOM^+^ interneurons of the spinal dorsal horn, and the in situ PLA assay was performed 24 h after CFA injection. Compared to saline control, CFA injection significantly decreased the PLA signals (GlyR α1 + PY20) in spinal SOM^+^ interneurons (Fig. 3d), suggesting a reduction of GlyR α1 tyrosine phosphorylation. Knockdown of PTP1B effectively attenuated the reduction of PLA signal induced by CFA (Fig. 3d), suggesting that PTP1B was essential for the tissue injury-induced dephosphorylation of GlyR α1. In support of the critical role of PTP1B in CFA-induced dephosphorylation of GlyR α1, electrophysiological recordings on spinal SOM^+^ interneurons showed that CFA sharply decreased the amplitudes of GlyR-mIPSCs (Fig. 3e), meanwhile the frequency of GlyR-mIPSCs, a readout of presynaptic release probability, was decreased (Fig. 3e). Selective knockdown of PTP1B in spinal SOM^+^ interneurons and extracellular perfusion of PTP1B inhibitor XXII effectively restored the peripheral injury-induced reduction of GlyR-mIPSC amplitudes, without significantly affecting the frequencies of GlyR-mIPSCs (Fig. 3e), suggesting that PTP1B was required for the reduction of postsynaptic GlyR function or number induced by peripheral inflammatory injury.

To investigate the potential contribution of PTP1B to CFA-induced hyperexcitability of SOM^+^ neurons in the spinal dorsal horn, we elicited the action potentials (APs) by depolarizing current injections under the whole-cell current-clamp configuration. Compared to saline control, CFA injection led to a notable increase in the AP firing frequencies of SOM^+^ interneurons (Fig. 3f), which were accompanied by reduced rheobase (Fig. 3g), and shifted resting membrane potential (Fig. 3h). However, these effects of CFA were almost completely abolished by PTP1B knockdown or PTP1B inhibitor. Following the inhibition of glycinergic transmission by GlyR antagonist strychnine, PTP1B knockdown did not affect AP firings of spinal SOM^+^ neurons from inflamed mice (Suppl. Fig. 3b). These results suggested that PTP1B-mediated glycinergic disinhibition participated in the hyperexcitability of spinal SOM^+^ neurons.

The low-threshold Aβ fiber input has been shown to elicit both excitation and feedforward inhibition of SOM^+^ interneurons (Suppl. Fig. 3a). Glycinergic disinhibition in the context of chronic pain conditions is proposed as a key mechanism by which Aβ afferents drive the excitatory output from SOM^+^ interneurons to spinal cord nociceptive circuits, leading to mechanical allodynia^5,10^. It has also been demonstrated that the electrical stimulation of dorsal roots at Aβ fiber strength (25 μA) evoked AP discharge in 55% (11/20) of SOM^+^ neurons in CFA-injection mice (Suppl. Fig. 3c), while only 5% (1 of 20) of SOM^+^ interneurons fired APs in response to Aβ fiber activation in naïve mice (Suppl. Fig. 3c). Next, we selectively knocked down PTP1B in spinal SOM^+^ neurons from inflamed mice. Only 2 of 20 recorded neurons (10%) exhibited AP firings in response to Aβ-fiber stimulation (Suppl. Fig. 3c), the proportion significantly lower than that observed in spinal SOM^+^ neurons from CFA-injected mice. Following the inhibition of glycinergic transmission by GlyR antagonist strychnine, knockdown of PTP1B in spinal SOM^+^ neurons did not decrease the proportion of AP firings from inflamed mice (Suppl. Fig. 3b). These results demonstrated that glycinergic disinhibition induced by the enhanced expression of PTP1B after peripheral inflammatory injury partially contributed to the excitatory output from SOM^+^ interneurons.

### PTP1B knockdown in spinal SOM^+^ neurons or intrathecal injection of PTP1B inhibitor XXII inhibited inflammatory mechanical pain

**Fig. 4:**
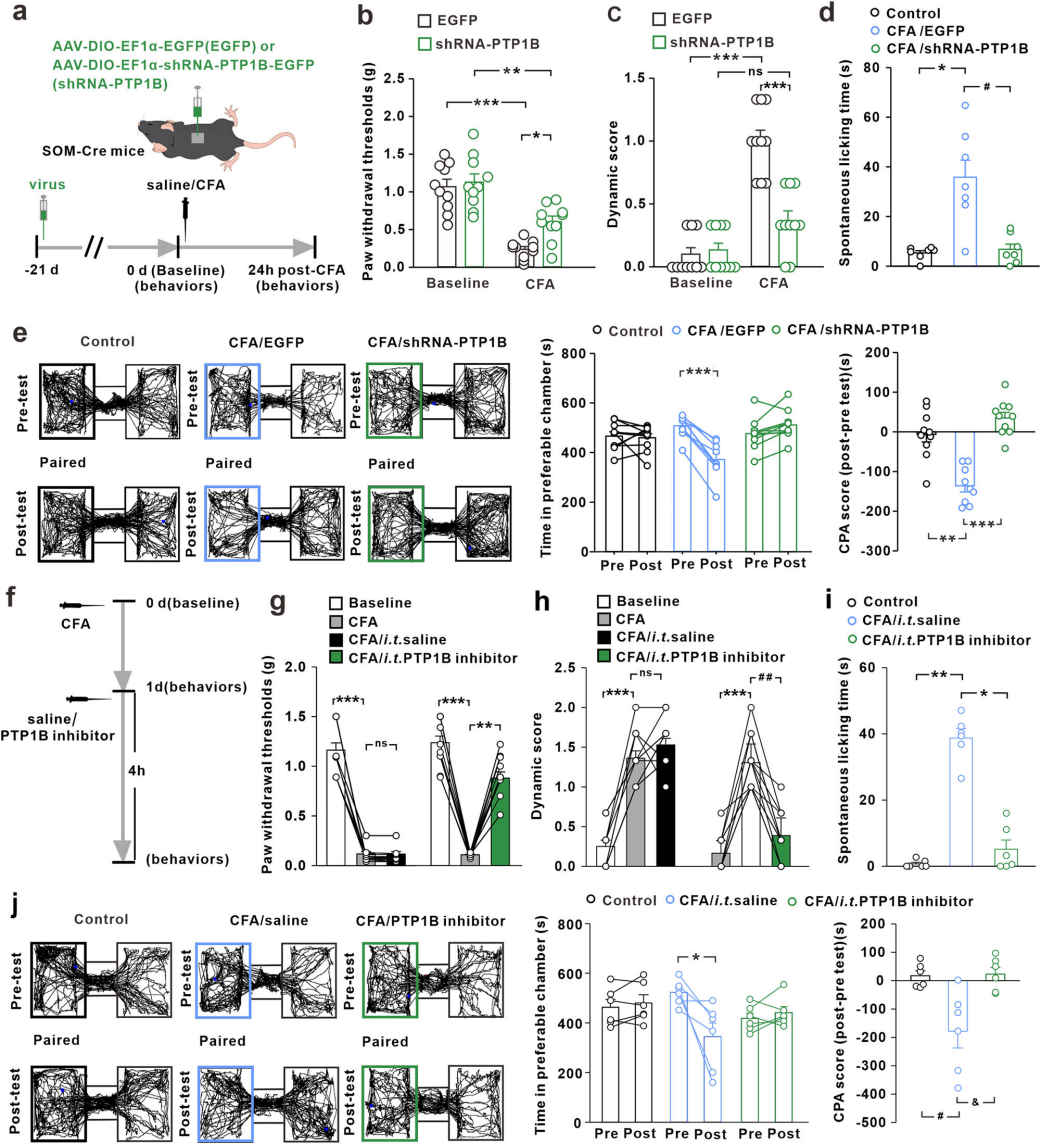
Knockdown of PTP1B in spinal SOM^+^ neurons or intrathecal administration of PTP1B inhibitor XXII inhibited inflammatory mechanical pain. **a** Schematic of intraspinal viral injection and experimental schedule in SOM-Cre mice. **b**, **c** von Frey thresholds (**b**; *P = 0.028, **P = 0.001, ***P < 0.001) and brush-evoked pain responses (**c**; ***P < 0.001, ^ns^*P* = 0.132) at 24 h after intradermal (i.d.) saline or CFA injection in SOM-Cre mice expressing EGFP or shRNA-PTP1B. Kruskal–Wallis test. *n* = 10 mice/group. **d** Spontaneous licking behaviors of mice at 24 h after intradermal (i.d.) injection of saline or CFA in SOM-Cre mice expressing EGFP or shRNA-PTP1B. *P = 0.016, ^#^*P* = 0.024 (Kruskal–Wallis test), *n* = 7 mice per group. **e** Brushing evoked conditioned place aversion (CPA) in inflamed SOM-Cre mice with spinal SOM^+^ interneurons expressing EGFP but not shRNA-PTP1B. ***P < 0.001 (t_8_ = 8.774, paired Student’s *t*-test), **P = 0.01, ***P < 0.001 (Kruskal–Wallis test). *n* = 10 mice per group. **f** Experimental schedule in C57BL/6J mice. **g**, **h** Intrathecal (i.t.) application of PTP1B inhibitor XXII (10 nmol) for 4 h attenuated punctate mechanical allodynia (**g**) and dynamic mechanical allodynia (**h**) in CFA mice. ***P < 0.001, ^##^*P* = 0.001, **P = 0.006, ^ns^*P* = 1.00 (Kruskal–Wallis test**)**^.^ *n* = 12 mice per group. **i** Spontaneous licking activities of mice at 4 h after intradermal (i.d.) saline or PTP1B inhibitor XXII (10 nmol) in CFA mice. **P = 0.002, *P = 0.034 (Kruskal–Wallis test). *n* = 6 mice per group. **j** Intrathecal (i.t.) application of PTP1B inhibitor XXII (10 nmol) alleviated brushing-evoked CPA in inflamed mice. ***P = 0.03 (t_5_ = 3.017, paired Student’s *t*-test), ^#^*P* = 0.039, ^&^*P* = 0.039 (Kruskal–Wallis test). *n* = 6 mice/ group. All data were shown as mean ± SEM.

Given that PTP1B was essential for the CFA-induced glycinergic disinhibition, we tested whether knockdown of PTP1B in SOM^+^ interneurons was able to generate analgesic action on inflammatory pain. EGFP and shRNA-PTP1B were pre-expressed into SOM^+^ interneurons before CFA injection, and a series of behavioral assessments were conducted (Fig. 4a). The von Frey test showed that CFA injection induced a significant reduction in reflexive withdrawal thresholds in both the EGFP and shRNA-PTP1B groups (Fig. 4b). Nonetheless, the withdrawal thresholds in the shRNA-PTP1B group were significantly higher than those in the EGFP group (Fig. 4b), suggesting that shRNA-PTP1B alleviated CFA-induced static mechanical allodynia, albeit to a modest extent. The CFA-induced dynamic allodynia was also inhibited by shRNA-PTP1B, manifested as reduced dynamic score to the brush stimuli (Fig. 4c). Moreover, knockdown of PTP1B in SOM^+^ interneurons was able to reverse the increased spontaneous licking behaviors in CFA-injected freely moving mice (Fig. 4d). No alterations in the locomotor activity were observed after CFA injection or shRNA-PTP1B expression, as evidenced by the comparable total traveling distance (Suppl. Fig. 4a), The CFA injection also led to marked heat and cold hypersensitivities (Suppl. Fig. 4b, c), but the expression of shRNA-PTP1B failed to ameliorate the hypersensitivity induced by CFA (Suppl. Fig. 4b, c). These results suggested that PTP1B in SOM^+^ interneurons specifically contributed to mechanical hypersensitivity.

To test the influence of PTP1B on pain aversion, we conducted a conditioned place aversion (CPA) assay. CFA-injected mice spent less time in the chamber where they suffered from repetitive gentle brushings, suggesting that CFA injection enabled the light touch to recruit supraspinal circuits for aversive pain memory (Fig. 4e). Prior expression of shRNA-PTP1B, however, significantly reduced avoidance of the brush-paired chamber (Fig. 4e), suggesting that knockdown of PTP1B in SOM^+^ interneurons was able to attenuate the tonic aversive state of inflamed mice.

Next, we investigated the effect of PTP1B inhibitor XXII on the inflammatory pain. Intrathecal injection of PTP1B inhibitor XXII reversed the reduction of the paw withdrawal thresholds induced by CFA (Fig. 4g). To further assess dynamic mechanical allodynia, we applied gentle brush stroking to the plantar surfaces of the hindpaws and observed that PTP1B inhibitor XXII also attenuated the dynamic inflammatory allodynia (Fig. 4h). Moreover, intrathecal administration of PTP1B inhibitor XXII reversed the increased spontaneous licking behaviors in CFA-injected mice (Fig. 4i). The CPA assays showed that the gentle brush stroking was aversive to CFA-injected mice because they spent less time in the chamber where the repetitive brushings were applied. Prior delivery of PTP1B inhibitor XXII reduced avoidance of the brush-paired chamber (Fig. 4j), suggesting that PTP1B inhibitor XXII was sufficient to attenuate the light touch-evoked unpleasant aversive feeling of inflamed mice.

Given that glycinergic disinhibition plays a major role in neuropathic pain^22,23^, we investigated the role of PTP1B in glycinergic disinhibition and mechanical allodynia in the spared nerve injury (SNI) model. Along with the decline in PWTs and the increase in dynamic scores at day 7 after SNI (Suppl. Fig. 5a, b), the total expression of PTP1B in EGFP-labeled SOM^+^ interneurons of the spinal dorsal horn significantly increased (Suppl. Fig. 5c). Knockdown of PTP1B in spinal SOM^+^ interneurons attenuated the peripheral nerve injury-induced glycinergic disinhibition (Suppl. Fig. 5d). In addition, prior knockdown of PTP1B in SOM^+^ interneurons significantly alleviated the static mechanical allodynia (Suppl. Fig. 5e), dynamic mechanical allodynia (Suppl. Fig. 5f), spontaneous licking behaviors (Suppl. Fig. 5g) and light touch-evoked unpleasant aversive feeling (Suppl. Fig. 5h), suggesting that the enhancement of PTP1B expression in SOM^+^ interneurons contributed to glycinergic disinhibition and mechanical allodynia in neuropathic pain model of mice.

### PTP1B and GPR39 competed for GlyR α1 binding

**Fig. 5:**
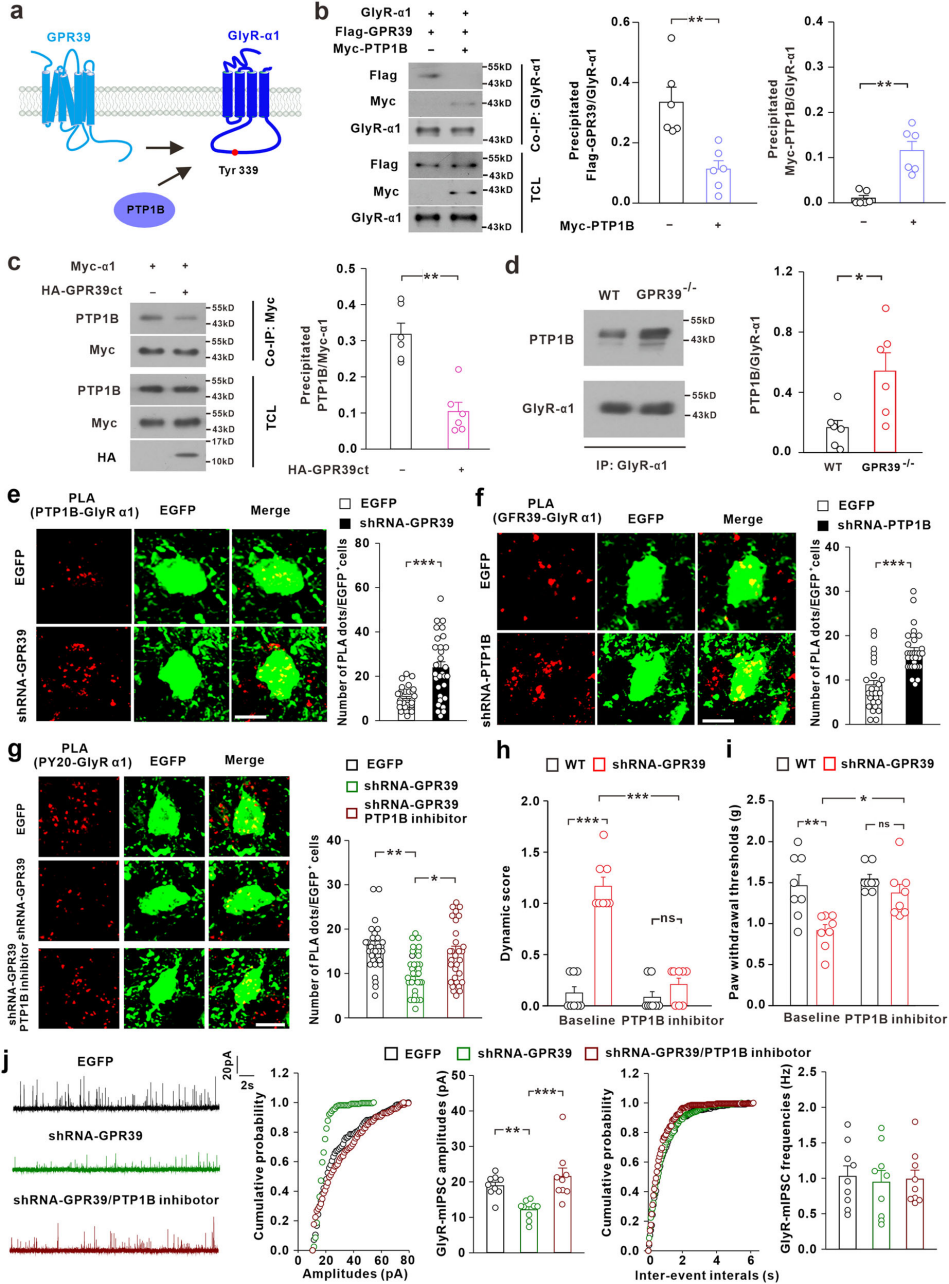
PTP1B and GPR39 competed for GlyR α1 binding. **a** Schematic diagram of GPR39 and PTP1B that competitively bound to GlyR α1 and determined Tyr^339^ phosphorylation. **b** Effects of Myc-tagged PTP1B(1-321/D181A) (Myc-PTP1B) on Flag-GPR39 interaction with GlyR α1 (left). Graphs summarized the ratio of precipitated Flag-GPR39 to GlyR α1 (middle; **P = 0.002, Mann–Whitney *U*-test) or the ratio of precipitated Myc-PTP1B to GlyR α1 (right; **P = 0.002, Mann–Whitney *U*-test). *n* = 6 experiments. **c** Effects of HA-GPR39ct on PTP1B/Myc-α1 interaction in HEK293T cells. **P = 0.002 (Mann–Whitney *U*-test). *n* = 6 experiments. **d** GlyR α1 immunoprecipitates from spinal dorsal horn of WT and GPR39^−/−^ mice were immunoblotted for PTP1B and GlyR α1. *P = 0.015 (Mann–Whitney *U*- test), *n* = 6 experiments. **e** PLA detection of PTP1B-GlyR α1 complex in spinal SOM^+^ neurons expressing EGFP or shRNA-GPR39. ***P < 0.001 (Mann–Whitney *U*-test). *n* = 30 neurons from three mice per group. Scale bar, 5 μm. **f** PLA detection of GPR39-GlyR α1 complex in spinal SOM^+^ neurons expressing EGFP or shRNA-PTP1B. ***P < 0.001 (Mann–Whitney *U*-test). *n* = 29 neurons (EGFP) and 31 neurons (shRNA-PTP1B) from 3 mice per group. Scale bar, 5 μm. **g** PLA assay of GlyR α1 tyrosine phosphorylation in SOM^+^ interneurons expressing EGFP or shRNA-GPR39. PTP1B inhibitor (10 nmol) was spinally injected for 4 h before PLA assay. *P = 0.0228, **P = 0.0015 (one-way ANOVA and post hoc Bonferroni test). *n* = 30 neurons from three mice per group. Scale bar, 5 μm. **h**, **i** Responses of WT and GPR39^−/−^ mice to paintbrush (**h**; ***P < 0.001, ^ns^*P* = 1.00) and von Frey stimulation (**i**; **P = 0.003, *P = 0.015, ^ns^*P* = 1.00) before (baseline) and 4 h after intrathecal PTP1B inhibitor XXII (10 nmol) injection. One-way ANOVA and post hoc Bonferroni test, *n* = 8 mice/group. **j** Effects of extracellular perfusion of PTP1B inhibitor XXII (1 μM) on GlyR-mIPSCs on SOM^+^ neurons expressing EGFP or shRNA^-^GPR39 in SOM-tdTomato mice. **P = 0.017, ***P < 0.001 (one-way ANOVA and post hoc Bonferroni test), *n* = 9 neurons from three mice per group. All data were shown as mean ± SEM.

GPR39 interacts with the intracellular large loop (ILL) of GlyR α1^10^. The GlyR α1-ILL was also responsible for PTP1B binding (Fig. 1e). We hypothesized that GPR39 might compete with PTP1B for GlyR α1 binding (Fig. 5a). To test this, we transfected the substrate trapping mutant of PTP1B, PTP1B(1-321/D181A), along with GlyR α1 and Flag-GPR39 in HEK293T cells. Co-immunoprecipitation analysis showed that the interaction of PTP1B(1-321/D181A) with GlyR α1 was paralleled by the disengagement of Flag-GPR39, suggesting that PTP1B could mutually block GPR39 incorporation into GlyR α1 complex (Fig. 5b).

The extreme carboxyl tail of GPR39 (GPR39ct) is responsible for GlyR α1 binding^10^. We found that expression of GPR39ct was sufficient to disrupt PTP1B interaction with Myc-α1 (Fig. 5c). In support of the competitive model, the interaction of GPR39 with GlyR α1 was sharply increased in GPR39^−/−^mice relative to WT controls (Fig. 5d).

To investigate whether PTP1B and GPR39 competitively bind to GlyR α1 in spinal SOM^+^ interneurons, EGFP or shRNA-GPR39 was transfected into SOM^+^ interneurons of the spinal dorsal horn and in situ PLA assay was performed. Compared to EGFP, shRNA-GPR39 significantly increased the PLA signals for the PTP1B-GlyR α1 interaction (Fig. 5e). Similarly, knockdown of PTP1B in SOM^+^ interneurons also markedly enhanced the interaction of GlyR α1 with GPR39 (Fig. 5f).

Given that GPR39 and PTP1B competitively bound‌ to GlyR α1, we tested if knockdown of GPR39 led to PTP1B-mediated dephosphorylation of GlyR α1. PLA assay showed that expression of shRNA-GPR39 in SOM^+^ interneurons significantly decreased the PLA (GlyR α1 + PY20) puncta when compared with EGFP (Fig. 5g). The decreased tyrosine phosphorylation of GlyR α1 induced by shRNA-GPR39 was completely abolished in the presence of PTP1B inhibitor XXII (Fig. 5g). Consistent with the effect of XXII on dephosphorylation of GlyR α1 in GPR39 knockdown mice, XXII alleviated the dynamic (Fig. 5h) and static mechanical allodynia (Fig. 5i). Moreover, GPR39 knockdown led to a significant decrease in glycinergic synaptic transmission in spinal SOM^+^ interneurons, which could be reversed by extracellular perfusion of XXII (Fig. 5j).

### GPR39 inhibited the interaction between GlyR α1 and PTP1B in CFA-injected mice

**Fig. 6:**
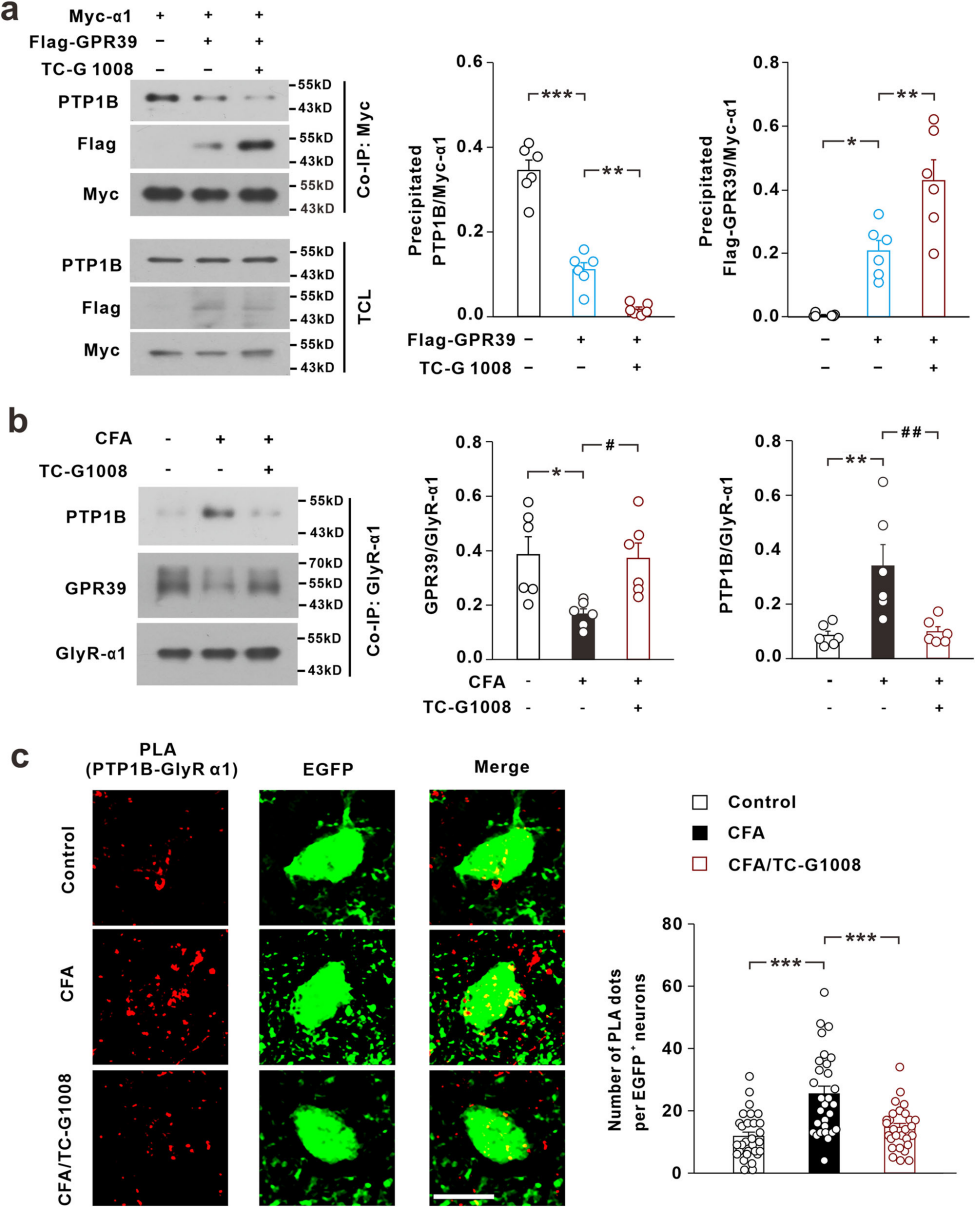
Activation of GPR39 enhanced GPR39-GlyR α1 interaction and disrupted PTP1B from GlyR α1. **a** TC-G 1008 (50 pmol) enhanced the ability of Flag-GPR39 to displace PTP1B from Myc-α1 complex in HEK293T cells (left). Graphs summarized the ratio of precipitated PTP1B to Myc-α1 (middle; **P = 0.005, ***P < 0.001) or the ratio of precipitated Flag-GPR39 to Myc-α1 (right; *P = 0.013, **P = 0.007). One-way ANOVA and post hoc Bonferroni test. *n* = 6 experiments. Total cell lysates (TCL) were immunoblotted for PTP1B, Flag and Myc. **b** Changes of GlyR α1 complex with GPR39 and PTP1B after intrathecal TC-G 1008 (50 pmol) treatment for 30 min in CFA-injected mice (left). Graphs showed the ratio of precipitated GPR39 to GlyR α1 (middle; ^#^*P* = 0.038, *P = 0.026) or the ratio of precipitated PTP1B to GlyR α1 (right; **P = 0.005, ^##^*P* = 0.008). One-way ANOVA and post hoc Bonferroni test, *n* = 6 experiments. **c** PLA dete**c**tion of PTP1B-GlyR α1 complex in spinal sections from control or CFA-injected SOM-Cre mice that were pretreated with intrathecal TC-G 1008 (50 pmol). ***P < 0.001 (one-way ANOVA and post hoc Bonferroni test). *n* = 29 neurons from three mice per group. Scale bar, 10 μm. All data were shown as mean ± SEM.

GPR39 agonist TC-G 1008 potentiates the glycinergic synaptic transmission on spinal SOM^+^ neurons of an inflamed mouse^10^. We want to know if the effect of TC-G 1008 was related to the alteration in the interactions of GlyR α1. We transfected Myc-tagged GlyR α1 (Myc-α1) along with Flag-GPR39 in HEK293T cells. In the absence of GPR39 agonist TC-G 1008, the expression of Flag-GPR39 was able to enhance the incorporation of Flag-GPR39 into Myc-α1 complex, which was coincident with the dissociation of PTP1B from Myc-α1 complex (Fig. 6a). In the presence of GPR39 agonist, the expression of Flag-GPR39 led to an additional increase in the interactions of GPR39 with Myc-α1, which was accompanied by additional declines of Myc-α1 complex with PTP1B (Fig. 6a), suggesting that activation of GPR39 increased GPR39 interaction with GlyR α1 by disrupting PTP1B from GlyR α1.

In the inflammatory pain model of mice, subcutaneous injection of CFA caused a significant decline in the interaction of GPR39 with GlyR α1, which was coincident with an increased incorporation of PTP1B into the GlyR α1 complex (Fig. 6b). This effect of CFA was significantly reversed by intrathecal application of TC-G 1008 (Fig. 6b), suggesting that activation of GPR39 enhanced GlyR α1-GPR39 interaction by displacing PTP1B from GlyR α1 in inflamed mice. In line with this observation, intrathecal application of TC-G 1008 markedly reduced PTP1B incorporation into GlyR α1 in spinal SOM^+^ interneurons of inflamed mice (Fig. 6c).

Emerging evidence indicates that GPR39 is expressed in the skin and dorsal root ganglia (DRG)^24,25^. However, our previous study showed that GPR39 signals were not coincident with the central terminals of DRG neurons positive for calcitonin gene-related peptide (CGRP^+^) and isolectin B4 (IB4^+^)^10^. To determine whether the antinociceptive effect of the GPR39 agonist TC-G 1008 was specifically mediated by GlyR modulation in spinal SOM^+^ interneurons, we administered TC-G 1008 intrathecally or subcutaneously. Neither route affected hindpaw volume in CFA-injected mice (Suppl. Fig. 6). These findings suggested that TC-G 1008 alleviated mechanical allodynia specifically by modulating glycinergic transmission in spinal SOM^+^ interneurons, rather than through modulation of peripheral inflammatory processes.

## Discussion

G protein-coupled receptor 39 (GPR39) has been found to maintain basic glycinergic inhibition by interacting with GlyR α1 in spinal somatostatin-positive (SOM^+^) interneurons^10^. In this study, we found that GlyR α1 was subjected to negative regulation by PTP1B. Because PTP1B and GPR39 competed for GlyR α1 binding, peripheral inflammation-induced hyperexpression of PTP1B disrupted the GPR39-GlyR α1 interaction and contributed to glycinergic disinhibition and mechanical pain hypersensitivity. Pharmacological activation of GPR39 enhanced GlyR α1-GPR39 interaction and led to the removal of PTP1B from GlyR α1. These findings provided a perspective for understanding the molecular mechanisms of the spinal glycinergic disinhibition following peripheral inflammation and the analgesic action of GPR39 agonist TC-G 1008.

Convincing evidences have shown the crucial role of spinal SOM^+^ neurons in transmitting the mechanical allodynia^4,5,10,26^. The genetic ablation of spinal SOM^+^ neurons selectively blocks the mechanical allodynia in both inflammatory and neuropathic pain conditions^4,5^. We observed that PTP1B was abundantly expressed in SOM^+^ neurons. The expression level of PTP1B in SOM^+^ neurons was significantly upregulated after peripheral inflammation. Correspondingly, knockdown of PTP1B of spinal SOM^+^ neurons selectively alleviated peripheral inflammation-induced mechanical pain without effects on the thermal and cold sensitivities, suggesting that PTP1B was an attractive target for treatment of inflammatory mechanical pain.

PTP1B is a ubiquitously expressed tyrosine phosphatase in the central nervous system^27–31^. Recent studies indicated the localization of PTP1B at the excitatory postsynaptic density in the spinal dorsal horn^32^. Here, we found that PTP1B was also presented at gephyrin^+^ inhibitory postsynaptic sites of SOM^+^ neurons, suggesting a possible role in the modification of inhibitory postsynaptic function. Electrophysiological recordings in spinal slices and HEK293T cells demonstrated that PTP1B inhibition significantly enhanced the postsynaptic GlyR function or number, albeit to a modest extent. The brain-derived neurotrophic factor receptor TrkB, glutamate receptor mGluR5, synapse-associated protein 102 and Src-family protein tyrosine kinase have been reported to be the substrates of PTP1B^29,32–34^. In several pathological conditions, the hyperactivation or hyperexpression of PTP1B enhances dephosphorylation of these substrates to alter the neuronal excitability^28,35–37^. A recent study has shown that spinal PTP1B augmented the synaptic transmission mediated by GluN2B subunit-containing NMDA receptors^32^. In the present study, we identified that GlyR α1, the major player in glycinergic transmission in adult spinal dorsal horn^38,39^, was a significant substrate of PTP1B. The dephosphorylation of GlyR α1 by PTP1B negatively regulated glycinergic transmission. These results suggested that PTP1B was able to modify synaptic transmission through both postsynaptic GlyR α1 and NMDAR in the spinal dorsal horn.

The activity-dependent regulations of GlyRs number on the plasma membrane and postsynaptic sites are important ways to modulate inhibitory synaptic strength^17,40^. The intracellular trafficking and synaptic localization of GlyRs is precisely regulated by posttranslational modifications. There is growing evidence that the phosphorylation levels of GlyR regulate receptor exocytosis, endocytosis and lateral diffusion to modification of glycinergic transmission^17,41^. The tyrosine residue (Tyr339) within the intracellular large loop of GlyR α1 has been shown as a unique site for tyrosine phosphorylation and implicated in the regulation of GlyRs function^10^. In this study, we demonstrated that the PTP1B-mediated dephosphorylation negatively regulated glycinergic transmission. Presumably, the tyrosine dephosphorylation might reduce the number of receptors at inhibitory synapses by weakening the synaptic localization or lateral diffusion of GlyR α1. The precise mechanism underlying this modification requires further investigation.

GPR39 belongs to the ghrelin/neurotensin receptor subfamily. The dysregulation of GPR39 has been implicated in the pathogenesis of multiple neurological disorders, including chronic pain^15,16,42,43^. Our previous study found that GPR39 maintains basal glycinergic inhibition by interacting with GlyR α1 subunit, and that pharmacological activation of GPR39 potentiates glycinergic transmission^10^, but it remains unknown how GPR39 regulates glycinergic inhibition. In the present study, we found that the binding of GPR39 to GlyR α1 competitively displaced PTP1B from GlyR α1. Intriguingly, GPR39 agonist TC-G 1008 markedly increased the GPR39-GlyR α1 interaction, while concurrently reducing the interaction of PTP1B-GlyR α1. In inflamed mice, intrathecal application of TC-G 1008 significantly enhanced GlyR α1-GPR39 interaction, which was accompanied by a decreased GlyR α1-GPR39 interaction. In aligned with the action of TC-G 1008 on GlyR α1 complex, TC-G 1008 potentiated glycinergic transmission on SOM^+^ neurons and attenuated mechanical pain in inflamed mice^10^. Given that PTP1B knockdown in spinal SOM^+^ neurons attenuated glycinergic disinhibition and mechanical pain in inflamed mice, the displacement of PTP1B by GPR39 from GlyR α1 accounted for the analgesic effect of GPR39 agonist TC-G 1008.

Taken together, the present study revealed an important role of PTP1B in the spinal glycinergic disinhibition, and provided evidence that GPR39 rescued the glycinergic disinhibition and attenuated mechanical pain by displacing PTP1B from GlyR α1.

## Materials and methods

### Animals

We have complied with all relevant ethical regulations for animal use. All experimental procedures received ethical approval from the Institutional Animal Care and Use Committee of Lanzhou University. The male C57BL/6 J mice (6-8 weeks) were obtained from the Experimental Animal Center of Lanzhou University (Approval No: SYXK(GAN)-2023-0004). The SOM-Cre mice (JAX 013044) and Ai14 reporter mice (JAX 007914) were obtained from the Jackson Laboratory. To label SOM^+^ neurons with tdTomato, the SOM-Cre mice were crossed with Ai14 reporter mice. The PTP1B knockout mice (KOCMP-21646) and GPR39 knockout mice (KOCMP-00773) were obtained from Cyagen (Suzhou, China). The mice were group-maintained with 3-5 per cage at a room temperature of 22–25 °C and 50% humidity on a 12 h light/dark cycle with available ad libitum water and food. Complete Freund’s Adjuvant (CFA; Sigma-Aldrich, #F5881, 20 μl) was unilaterally subcutaneously injected into the plantar surfaces of hindpaws to induce inflammatory pain.

### Virus and reagents

AAV2/9-EF1α-DIO-EGFP (titer: 1.2 × 10^13^ vg/ml), AAV2/9-EF1α-DIO-shRNA-PTP1B-EGFP (titer: 4.0 × 10^13^ vg/ml) and AAV2/9-EF1α-DIO-EGFP-shRNA-GPR39 (titer: 5.0 × 10^13^ vg/ml) were obtained from Sunbio (Shanghai, China). The sequences of shRNA-PTP1B and shRNA-GPR39 were 5’-GATCCAAAAAAGGAGGAACACA AGGCACATTGTCTCTTGAACAATGTGCCTTGTGTTCCTCC-3’and 5’-GATCCA AAAAAGGTGTACCTGATCATCTTTGTTCTCTTGAAACAAAGATGATCAGGT ACACC-3’, respectively.

The cDNAs encoding full-length mouse *Glra1* and human *Gpr39* in the pcDNA3.1 or pcDNA3.1-Flag vector was obtained from Youbio (Changsha, China). The site-directed mutagenesis was employed to generate GlyR α1(Y339F) (the numbering excludes the signal peptide sequence). The extreme carboxyl-terminal region (residues 344–453) of human *Gpr39* was PCR subcloned and ligated into the pcDNA3.1-HA vector. The intracellular large loop of human *Glra1* (residues 308–392) was also PCR subcloned and inserted into the pGEX-6p-1 vector. The rat full-length *Ptpn1* cDNA in the pcDNA3.1-Flag vector was a gift from Anna Huttenlocher (Addgene plasmid #35988). PTP1B(1-321/D181A) in pcDNA3.1-Myc-HisA was purchased from Genewiz (Suzhou, China).

TC-G 1008 (Sigma-Aldrich) and PTP1B inhibitor XXII (Millipore) were dissolved in dimethyl sulfoxide, which were diluted with saline or extracellular solution. Picrotoxic (Sigma-Aldrich), CNQX (Sigma-Aldrich), DAP-V (Sigma-Aldrich) and strychnine (Sigma-Aldrich) were dissolved in saline or extracellular solution.

### Intraspinal injection of the virus

Viral injection was performed using a brain stereotaxic apparatus^10,44,45^. In brief, Mice were anesthetized deeply with sodium pentobarbital (i.p., 60–90 mg/kg) and mounted on a stereotaxic apparatus. A heating pad was used to maintain the body temperature of mice. The spinal cord was exposed through a laminectomy. A glass pipette (tip diameter: 40 μm) filled with the virus was connected to a microsyringe pump (R- 480, RWD, Shenzhen, China). Each injection (300 nl, 50 nl/min) was performed at a depth of 0.2–0.3 mm from the dorsal surface of L4-L5 lumbar spinal cord. After each injection, a glass pipette was left in place for 5 min to avoid back-flow of virus. Finally, the muscle and skin were sutured. The mice were returned to their home cages after recovery.

### Behavioral experiments

All behavioral testing were performed blindly. The mice were placed in a behavioral device for 30 min on 3 consecutive days to acclimate to the testing environment.

The von Frey test was used to evaluate the punctate mechanical allodynia^17,45^. In brief, the plantar surfaces of hindpaws were stimulated perpendicularly with a series of von Frey filaments. The 50% paw withdrawal thresholds to von Frey stimuli were calculated using the up-down method^45,46^.

The dynamic mechanical allodynia was measured with a custom-made paintbrush (the length of 5 mm) to stroke the plantar surfaces of hindpaws from heel to toe at a velocity of about 2 cm/s^10,17^. The responses to the dynamic paintbrush stimuli were scored as follows: Score 0, a fast movement or lifting of the stimulated paw; Score 1, a sustained lifting (more than 2 s) or single flinching of the stimulated paw; Score 2, a strong lateral lifting of the stimulated paw or a startle-like jumping; Score 3, multiple flinching or licking of the stimulated paw^5,45^. The brush-evoked stimulation was repeated three times at an interval of more than 3 min, and the average scores were obtained.

For the Hargreaves test^17^, the mice were placed on a transparent glass plate, and a beam of light was applied to the plantar surfaces of hindpaws to measure the paw withdrawal latencies. A cut-off time of 10 s was set to prevent potential tissue damage.

For the measurement of cold sensitivity^10^, a drop of acetone was carefully applied to the plantar surfaces of the hindpaws through a syringe, without making direct contact with the skin. The initial 10-s activities were excluded, and the time during which the mice licked their stimulated hindpaws was recorded over the subsequent 60 s.

To assess the spontaneous pain behaviors, the mice were individually placed in a transparent plexiglas cage (18 × 18 × 22 cm). After 15 min of habituation, we videotaped and analyzed the total traveling distance and the time spent on licking the hindpaws over a 15-min period^10,45^.

The conditioned place aversion (CPA) test was performed in a custom-made apparatus consisting of two chambers (black and white chambers), which were interconnected with a central neutral corridor. On day 1, each mouse was placed into the central neutral corridor to freely explore the apparatus for 15 min. The time mice spent in the preference chamber was calculated by SuperMaze software (XinRun). The majority of mice usually exhibited a mild preference for the dark chamber. On day 2, the time spent in the preferred chamber was recorded as the pre-conditioning time. On Days 3 and 5, the mice were confined to their preferred chamber, and a custom-made paintbrush was utilized to stimulate the plantar surfaces of the hindpaws from heel to toe for 15 min with an interval of ~2 s. On Days 4 and 6, the mice were confined to their non-preferred chamber for 15 min. On day 7, the mice were placed into the neutral corridor and allowed to explore the entire apparatus freely for 15 min. The time spent in the brushing-paired chamber was recorded as the post-conditioning time. The CPA score was calculated by the post-conditioning time minus the pre-conditioning time^10,47^.

The hindpaws swelling of mice was quantified using a plethysmometer (PV-200, Techmen)^48,49^. Briefly, each hindpaw was gently immersed in the water-filled chamber of the device, and the resulting water displacement volume was recorded. Three measurements were performed for hindpaws, and then the average volume (ml) was calculated.

### Immunohistochemistry of free-floating slices

Mice were deeply anesthetized with sodium pentobarbital (i.p., 60–90 mg/kg) and perfused through the ascending aorta with 20 ml saline followed by 10 ml ice-cold paraformaldehyde (4%). The spinal cord was dissected and post-fixed in 4% paraformaldehyde for 2 h at 4°C. After cryoprotecting in 30% sucrose at 4 °C overnight, Transverse sections (40-μm thickness) of L4-L5 lumbar segments were cut on a vibratome stage (Leica, VT1200S). Spinal slices were blocked in PBS containing 10% normal goat serum (NGS) and 0.25% Triton X-100 at 4 °C overnight, followed by incubation with primary antibodies for 24–36 h at 4 °C^10,45^. After three washes with PBS, the slices were incubated with Alexa 488-, Cy3-, or Alexa 647-conjugated secondary antibodies (Invitrogen) for 2 h at room temperature. Fluorescence images were captured by a confocal laser scanning microscope (STELLARIS 5, Leica). The primary antibodies included rabbit anti-PTP1B antibody (1:400; #B0809, Calbiochem) and mouse anti-gephyrin antibody (1:200; Synaptic System; #147021).

### Proximity ligation assay

The transverse spinal sections (40-μm thickness) were prepared as described above. Fluorescence PLA was performed with Duolink in situ fluorescence kit (#DUO92001; #DUO92005; #DUO92008; Sigma-Aldrich) according to the manufacturer’s instructions^10,45,50,51^. Briefly, the slices were incubated with Antigen Retrieval Solution (#P0090; Beyotime) for 10 min at room temperature. After the antigen retrieval, the sections were permeabilized with PBS containing 0.25% Triton X-100 overnight, blocked for 1 h at 37 °C with the Duolink blocking solution, and incubated at 4 °C overnight with a mixture of rabbit anti-GPR39 (1:500; #NLS142; Novus Biologicals) and mouse anti-GlyR α1 antibody (1:200; #146111; Synaptic System), a mixture of rabbit anti-GlyR α1 (1:200; #146003; Synaptic System) and mouse phosphotyrosine (PY) antibody (1:200; #P4110; Sigma-Aldrich) or a mixture of mouse anti-PTP1B antibody (1:200; #PH01; Calbiochem) and rabbit anti-GlyR α1 (1:200; #146003; Synaptic System). The primary antibodies were diluted in 1× Duolink Antibody Diluent. Following two washes with 1× Wash Buffer A, the spinal slices were incubated with Duolink In Situ PLA probe anti-rabbit PLUS (1:5) and anti-mouse MINUS (1:5) at 37 °C for 60 min in the humidified incubator. The unbound PLA probes were washed out with 1× Wash Buffer A. The ligase was diluted (1:40) with 1× ligation buffer and incubated with the sections for 30 min at 37 °C in the humidified incubator. After two washes with 1× Wash Buffer A, the slices were incubated for 100 min at 37 °C with the polymerase diluted (1:80) in 1× amplification buffer in the darkened, humidified incubator. The sections were sequentially washed with 1× Wash Buffer B (2 × 10 min) and 0.01× Wash Buffer B (1 × 10 min). Fluorescence images were captured with a confocal laser scanning microscope (STELLARIS 5, Leica).

### Immunoprecipitation, GST pull-down assay, and Western blot

The mice were deeply anesthetized with sodium pentobarbital (i.p., 60–90 mg/kg). The L4-L5 lumbar segment of the spinal cord was isolated in ice-cold artificial cerebrospinal fluid (ACSF; 119.0 mM NaCl, 2.5 mM CaCl_2_, 2.5 mM KCl, 1.3 mM MgCl_2_, 1.0 mM NaH_2_PO_4_, 26 mM NaHCO_3_, and 11 mM D-glucose, pH 7.4, oxygenated with 95% O_2_ and 5% CO_2_). The lumbar spinal cord or HEK293T cells were homogenized in Radioimmunoprecipitation Assay Buffer (RIPA) containing 150 mM NaCl, 1 mM EDTA, 50 mM Tris∙HCl (pH 8.0), 1.0% NP-40, 0.1% SDS, 0.5% sodium deoxycholate and phosphatases/proteases inhibitor cocktail (#P9599, Sigma-Aldrich). After centrifugation at 14,000×*g* for 10 min, the supernatant was collected, and the protein concentration was measured with the Bicinchoninic Acid Assay Kit (#P0012S Beyotime). The supernatant was incubated with the primary antibody under gentle rotation at 4 °C overnight. The immune complexes were then incubated with protein A/G agarose beads at 4 °C for 4 h. After extensive washes with RIPA buffer, the immunoprecipitates were boiled in SDS sample buffer for 5 min and subsequently subjected to western blot analysis.

GST-α1-ILL protein was expressed in *Escherichia coli* BL21 cells and purified as previously described^10,17,45^. The GST-α1-ILL protein bound to glutathione agarose beads was incubated for 4 h at 4 ˚°C with the whole-cell lysates from HEK293T cells expressing Myc-PTP1B(1-321/D181A). Following centrifugation at 800×*g*, the beads were collected, washed with RIPA buffer, and boiled in SDS sample buffer for immunoblotting analysis.

The protein samples were separated by SDS-polyacrylamide gel electrophoresis and subsequently transferred to polyvinylidene difluoride (PVDF) membranes. Following blocking with 5% non-fat milk, the membranes were incubated with primary antibodies at 4 °C overnight and washed with PBST and then incubated with horseradish peroxidase-conjugated secondary antibodies for 1 h under gentle rotation at room temperature. The protein signals were detected using enhanced chemiluminescence. The primary antibodies used in the present study included the rabbit anti-GlyR α1 (1:200; #146 003; Synaptic System), mouse anti-PTP1B antibody (1:200; #PH01; Calbiochem), mouse anti-GST (1:20,000; #E0019; Anbo), mouse anti-Flag antibody (1:5000; #E0017; Anbo), mouse anti-Myc (1:600; #sc-40; Santa Cruz), rabbit anti-Myc antibody (1:1000; #ab9106; Abcam), mouse anti-HA antibody (1:1000; #66006-1-Ig; Proteintech), mouse anti-phosphotyrosine (clone PY20, 1:500; #P4110; Sigma-Aldrich), mouse anti-β-actin antibody (1:800; #A5316; Sigma-Aldrich) and rabbit anti-GPR39 antibody (1:200; #NLS142; Novus).

### Preparation of spinal slices and electrophysiological recordings

Electrophysiological recordings were performed blindly by the experimenters without knowledge of the manipulations that the mice had received. The spinal cord was dissected under sodium pentobarbital anesthesia (i.p., 60–90 mg/kg) and removed rapidly in ice-cold oxygenated (95% O_2_ + 5% CO_2_) sucrose solution (50 mM sucrose, 95 mM NaCl, 7 mM MgCl_2_, 1.8 mM KCl, 1.2 mM NaH_2_PO_4_, 0.5 mM CaCl_2_, 26 mM NaHCO_3_, 15 mM D-glucose, pH 7.4). Transverse slices (300-μm thickness) were cut on a vibratome (Leica, VT1200S), and then perfused at the speed of 2–4 ml/min in the recording chamber with oxygenated artificial cerebrospinal fluid (ACSF, in mM: 119.0 NaCl, 2.5 CaCl_2_, 2.5 KCl, 1.3 MgCl_2_, 1.0 NaH_2_PO_4_, 26 NaHCO_3_, and 11 D-glucose, pH 7.4) for at least 30 min at 32 °C before recordings. Whole-cell patch clamp recordings were performed on EGFP-labeled SOM^+^ neurons in the superficial dorsal corn of the spinal cord and HEK293T cells expressing EGFP. The SOM^+^ neurons in lamina II and the outer layers of lamina III were visualized and recorded with an Olympus BX51WIF microscope fitted with a 40× water immersion objective.

To record the miniature inhibitory postsynaptic currents (mIPSCs), the membrane potential was held at 0 mV with an Axon 700B Amplifier in the voltage clamp mode^10,17^. The glass electrodes had the resistance of 3–5 MΩ when filled with the internal solution (in mM: 110.0 Cs_2_SO_4_, 5.0 KCl, 2.0 MgCl_2_, 5.0 Na_2_-ATP, 0.5 Na-GTP, 20.0 HEPES, 0.6 EGTA, pH 7.25, 310–320 mOsm). The GlyR-mIPSCs were pharmacologically isolated by adding GABA_A_R antagonist bicuculline (10 μM), AMPAR antagonist CNQX (10 μM), NMDAR antagonist D-APV (50 μM) and tetrodotoxin (TTX, 1 μM) in the external solution. The GABA_A_R-mIPSCs were pharmacologically isolated by adding GlyR antagonist strychnine (2 μM), AMPAR antagonist CNQX (10 μM), NMDAR antagonist D-APV (50 μM) and tetrodotoxin (TTX, 1 μM) in the external solution. The cutoff amplitude of 10 pA and the average noise level of 5 pA were used for the identification and quantification of mIPSCs.

For action potentials recordings^10^, the internal solution contained: 135.0 mM K-gluconate, 3.0 mM KCl, 10.0 mM HEPES, 0.5 mM EGTA, 1.0 mM MgCl_2_, 4.0 mM Mg- ATP, and 0.5 mM Na-GTP (pH 7.2 adjusted with KOH, 290 to 300 mOsm). The resting membrane potential was measured immediately after the establishment of the whole-cell configuration. The action potentials firings were elicited by injecting depolarizing currents in 2-pA increments from 0 to 40 pA in the current-clamp mode. To record Aβ fiber-evoked AP firings, the dorsal roots were electrically stimulated at an intensity of 25 μA, and APs were recorded at resting membrane potentials.

HEK293T cells were transfected with the pcDNA3.1 vector encoding GlyR α1 and GlyR α1(Y339F). The cells were perfused (2–4 ml/min) at room temperature with external solution containing (in mM) 145.0 NaCl, 10.0 HEPES, 5.0 KCl, 2.0 CaCl_2_, 1.0 MgCl_2_ and 11.0 D-glucose (pH 7.3). The recording glass pipettes were filled with internal solution containing (in mM: 140.0 CsCl, 1.0 CaCl_2_, 2.0 MgCl_2_, 10.0 HEPES, 8.0 EGTA, 3.0 Na_2_-ATP, and 0.1 Na-GTP, pH 7.2, 295–305 mOsm). The membrane potential was held at −80 mV. the glycinergic currents mediated by GlyR α1 and GlyR α1(Y339F) were evoked by briefly puffing glycine (1 mM, 10 ms) onto the cells expressing EGFP through an electrically controlled microperfusion system (MP2-S, INBIO) at interval of 30 s^10,17^. The series and input resistances were monitored in real time throughout each experiment. The recordings were excluded if this resistance changed by more than 15%. Signals were filtered at 2 kHz, sampled at 10 kHz.

### Statistics and reproducibility

All experimental data were shown as mean ± SEM and analyzed blindly using SPSS 23.0. The sample sizes were chosen based on prior experience with similar experiments. The PLA puncta in spinal SOM^+^ interneurons were specifically quantified. Electrophysiological, western blot data, and immunofluorescence data were analyzed by Clampfit 8.0 software, National Institutes of Health ImageJ, and Image-Pro Plus 6.0 software, respectively. We performed the paired Student’s *t*-test, Mann–Whitney *U*-test, or Kruskal–Wallis test for two-group comparisons. One-way Analysis of variance (ANOVA) followed by a post hoc Bonferroni test was used to analyze the data between multiple groups. The data between multiple groups occurring over time were compared by the repeated-measurement ANOVA followed by Bonferroni post hoc tests. *P* < 0.05 was considered as the criterion for statistical significance.

### Reporting summary

Further information on research design is available in the Nature Portfolio Reporting Summary linked to this article.

## Supplementary information

Supplementary information

Description of Additional Supplementary Files

Dataset 1

Reporting summary

## Data Availability

All data needed to evaluate the conclusions in the paper are present in the manuscript and supplementary data (Excel).

## Code availability

No custom computer code was generated or used in this study. All data analyses were performed using commercially available software.
