# Supplementary material for: Enhanced expression of spinal PTP1B contributes to peripheral inflammation- induced glycinergic disinhibition and mechanical pain
Source: Commun Biol. 2026 May 20;9:1080. doi: 10.1038/s42003-026-10318-5 (PMC13458505; doi:10.1038/s42003-026-10318-5)
Supplement: Supplementary file 1 [file 42003_2026_10318_MOESM1_ESM.pdf]

Enhanced expression of spinal PTP1B contributes to peripheral inflammation-induced glycinergic disinhibition and mechanical pain

Hu-Hu Bai[&,1], Xue Bai[&,1], Xiao-Xue Liu[&,1], Yu-Bo Gao[1], Juan Li[1], Xu Yang[1], Jia-Ning Dang[1], Ying Wang[1], Yu-Xuan Zhang[1], Zhi-Yang Zhang[1], Qi Zhang[1], Yan-Ni Liu[1], Jiang-Ping Liu[2], Zhan-Wei Suo [1,*]

[1] Department of Molecular Pharmacology, School of Pharmacy, Lanzhou University, Lanzhou, Gansu, 730000, PR China

[2] NHC Key Laboratory of Diagnosis and Therapy of Gastrointestinal Tumor, Institute of Clinical Research and Translational Medicine, Gansu Provincial Hospital, Lanzhou, Gansu, 730000, China

***Corresponding author**, E-mail: suozhw@lzu.edu.cn (Zhan-Wei Suo)

[&] These authors contributed equally to this work.

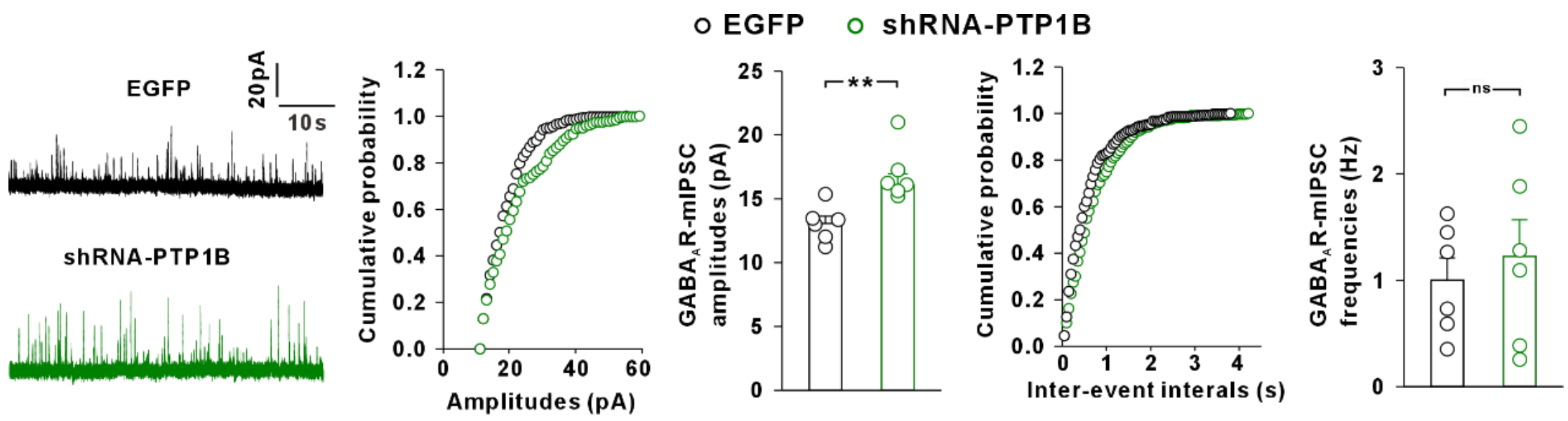

sFig. 1: Knockdown of PTP1B enhanced GABAergic transmission in spinal SOM$^+$ neurons. GABA$_A$R-mIPSCs were recorded on SOM$^+$ neurons expressing AAV-DIO-EF1α-EGFP (EGFP) or AAV-DIO-EF1α-shRNA-PTP1B (shRNA-PTP1B) in SOM-tdTomato mice. $^{**}P = 0.006$, $^{ns}P = 0.818$ (Mann-Whitney U test), n = 6 neurons from 3 mice per group.

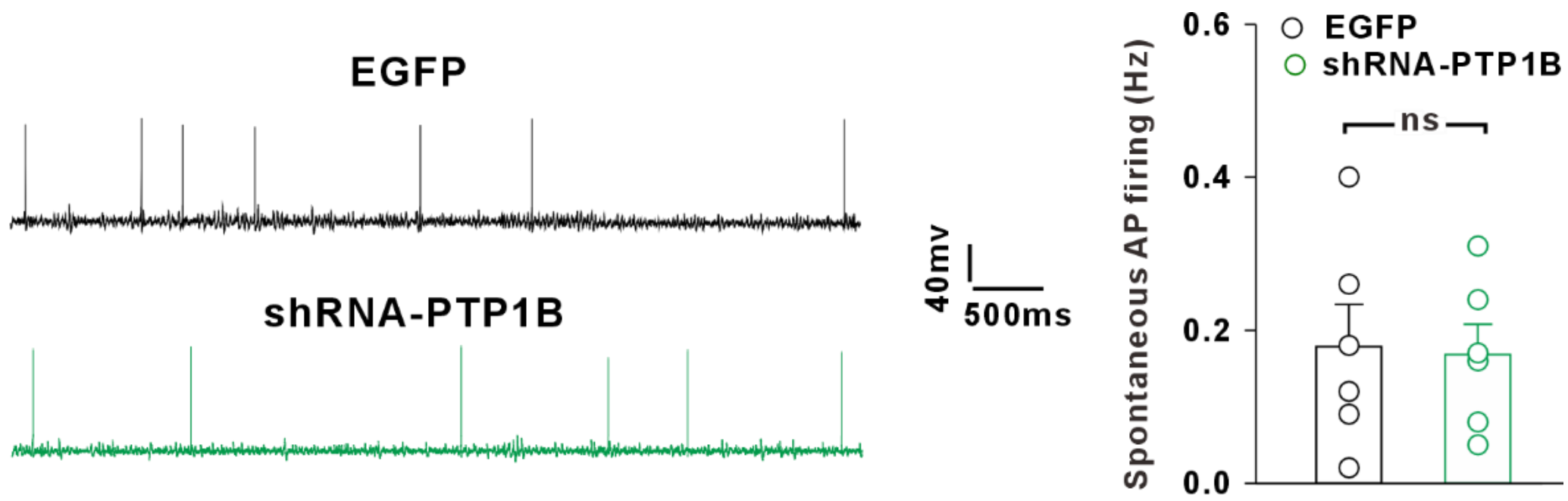

sFig. 2: Knockdown of PTP1B did not affect the activities of SOM-positive (SOM$^+$) neurons. Spontaneous AP firings were recorded on SOM$^+$ neurons expressing AAV-DIO-EF1α-EGFP (EGFP) or AAV-DIO-EF1α-shRNA-PTP1B (shRNA-PTP1B). n = 6 neurons from 3 mice per group.

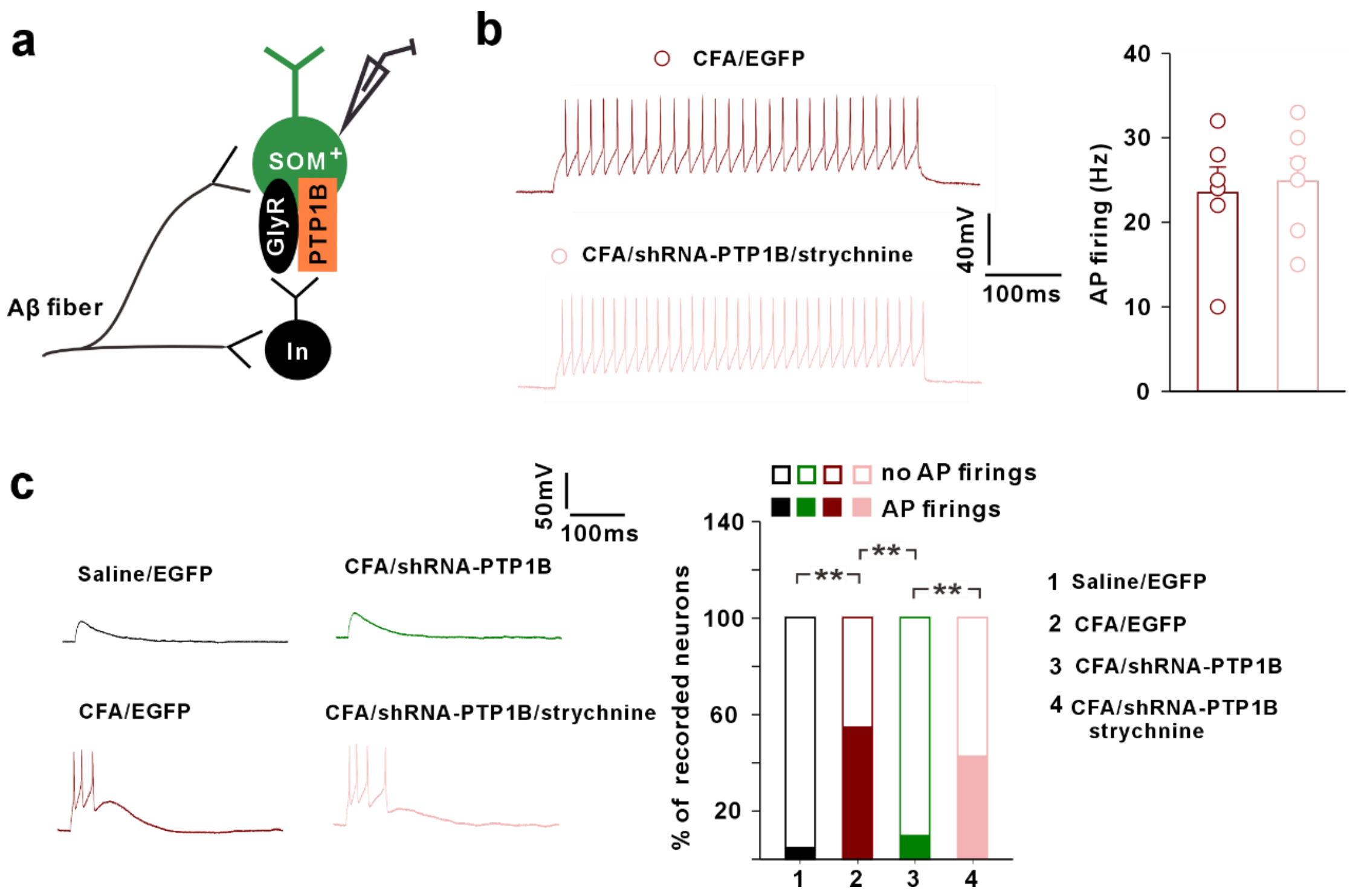

sFig. 3: Knockdown of PTP1B attenuated the peripheral inflammatory-induced hyperexcitability of somatostatin-positive (SOM$^+$) neurons. (a) Schematic illustration of Aβ fiber innervation of spinal SOM$^+$ neurons. In: inhibitory neurons. (**b**) Current-clamp recordings of AP firings elicited by depolarizing current injections in SOM$^+$ interneurons expressing EGFP or shRNA-PTP1B after saline or strychnine (2μM) perfusion in CFA-injection mice. **(c)** Current-clamp recordings of AP firings evoked by electrical stimulation of dorsal roots at Aβ fiber intensity (25 μA) at day 1 after CFA injection on SOM$^+$ neurons expressing AAV-DIO-EF1α-EGFP (EGFP) or AAV-DIO-EF1α-shRNA-PTP1B (shRNA-PTP1B). $**P < 0.01$ (Pearson's chi-square test).

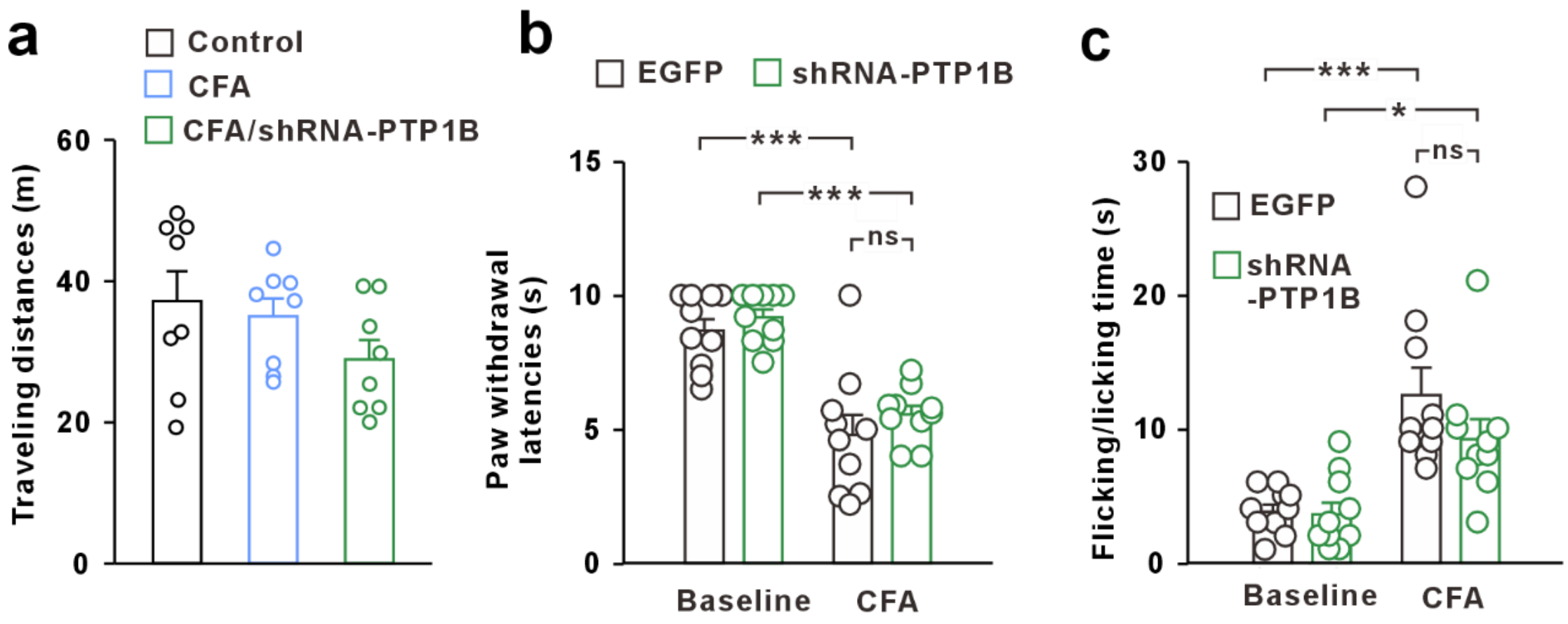

sFig. 4: Knockdown of PTP1B did not affect traveling distance (a), heat sensitivity (b) and cold sensitivity (c) before and after CFA injection in SOM-Cre mice expressing AAV-DIO-EF1α-EGFP (EGFP) or AAV-DIO-EF1α-shRNA-PTP1B (shRNA-PTP1B). b; $***P < 0.001$, $^{ns}P = 1.00$. c; $***P < 0.001$, $*P = 0.037$, $^{ns}P = 0.568$. Kruskal-Wallis test. n = 10 mice/group.

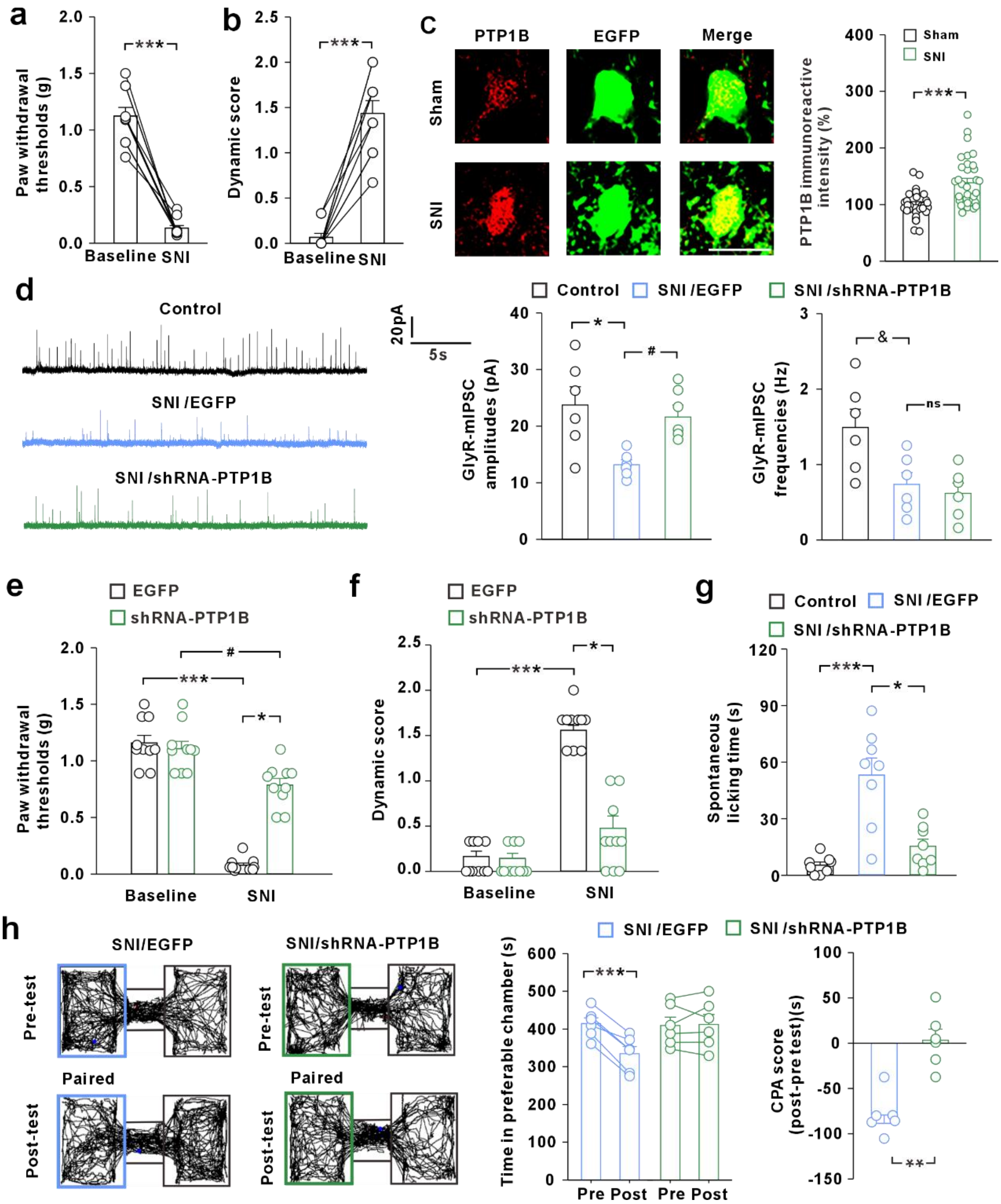

sFig. 5: Knockdown of PTP1B alleviated glycinergic disinhibition and mechanical pain evoked by spared nerve injury (SNI). (**a** and **b**) von Frey thresholds (a; ****P* < 0.001, t_9 = 10.995) and brush (b; ****P* < 0.001, t_9 = 9.473) before (baseline) and after SNI 7 days. Paired Student's *t* test. n = 10 mice/group. (**c**) Immunohistochemistry for PTP1B (red) in spinal SOM^+ neurons (green) at days 7 after SNI. ****P* < 0.001 (Mann-Whitney U test). n = 30 neurons from 3 mice per group. Scale bar, 10 µm. (**d**) GlyR-

mIPSCs recorded on SOM^+ neurons expressing EGFP or shRNA-GPR39 at days 7 after SNI in SOM-Cre mice. *P = 0.012, $^{\#}P$ = 0.031, $^{\&}P$ = 0.033, ^{ns}P = 1.00 (One-way ANOVA and post hoc Bonferroni test), n = 6 neurons from 3 mice per group. (**e** and **f**) von Frey thresholds (e; *P = 0.021, $^{\#}P$ = 0.037, ***P < 0.001) and brush-evoked pain responses (f; *P = 0.012, ***P < 0.001) of SOM-Cre mice expressing EGFP or shRNA-PTP1B at day 7 after SNI. Kruskal-Wallis test. n = 10 mice/group. (**g**) Spontaneous licking behaviors of SOM-Cre mice expressing EGFP or shRNA-PTP1B at days 7 after SNI. *P = 0.03, ***P < 0.001 (Kruskal-Wallis test), n = 6 mice per group. (**h**) Brushing evoked conditioned place aversion (CPA) in SNI mice whose spinal SOM^+ interneurons expressed EGFP rather than shRNA-PTP1B. ***P < 0.001 (t_5 = 8.652, paired Student's *t* test), **P = 0.004, (Mann-Whitney U test). n = 6 mice per group.

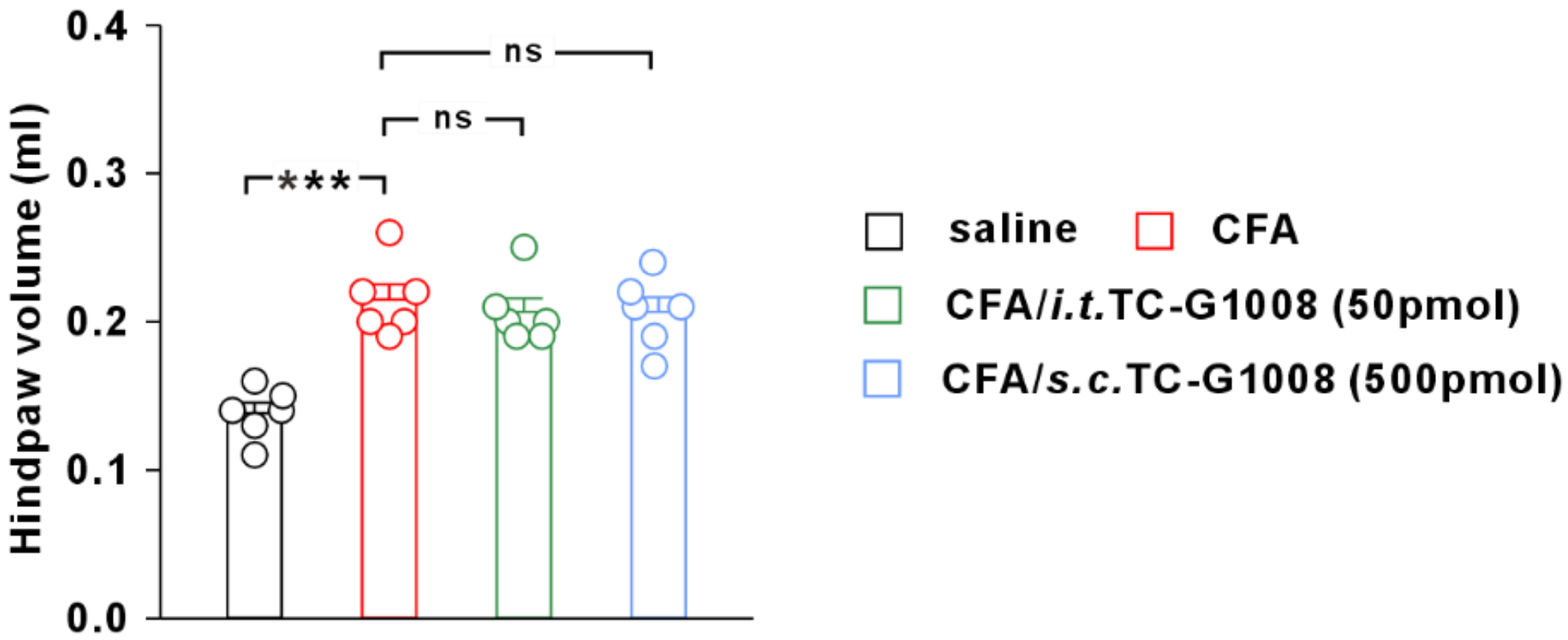

sFig. 6: Intrathecal (*i.t.* 50 pmol) and subcutaneous (*s.c.* 500 pmol) application of GPR39 agonist TC-G 1008 did not affect hindpaw volume of CFA-injection mice. ***$P < 0.001$, $^{ns}P = 1.00$. One-way ANOVA and post hoc Bonferroni test. n = 6 mice/group.

Original image of Western blot

Fig. 1a

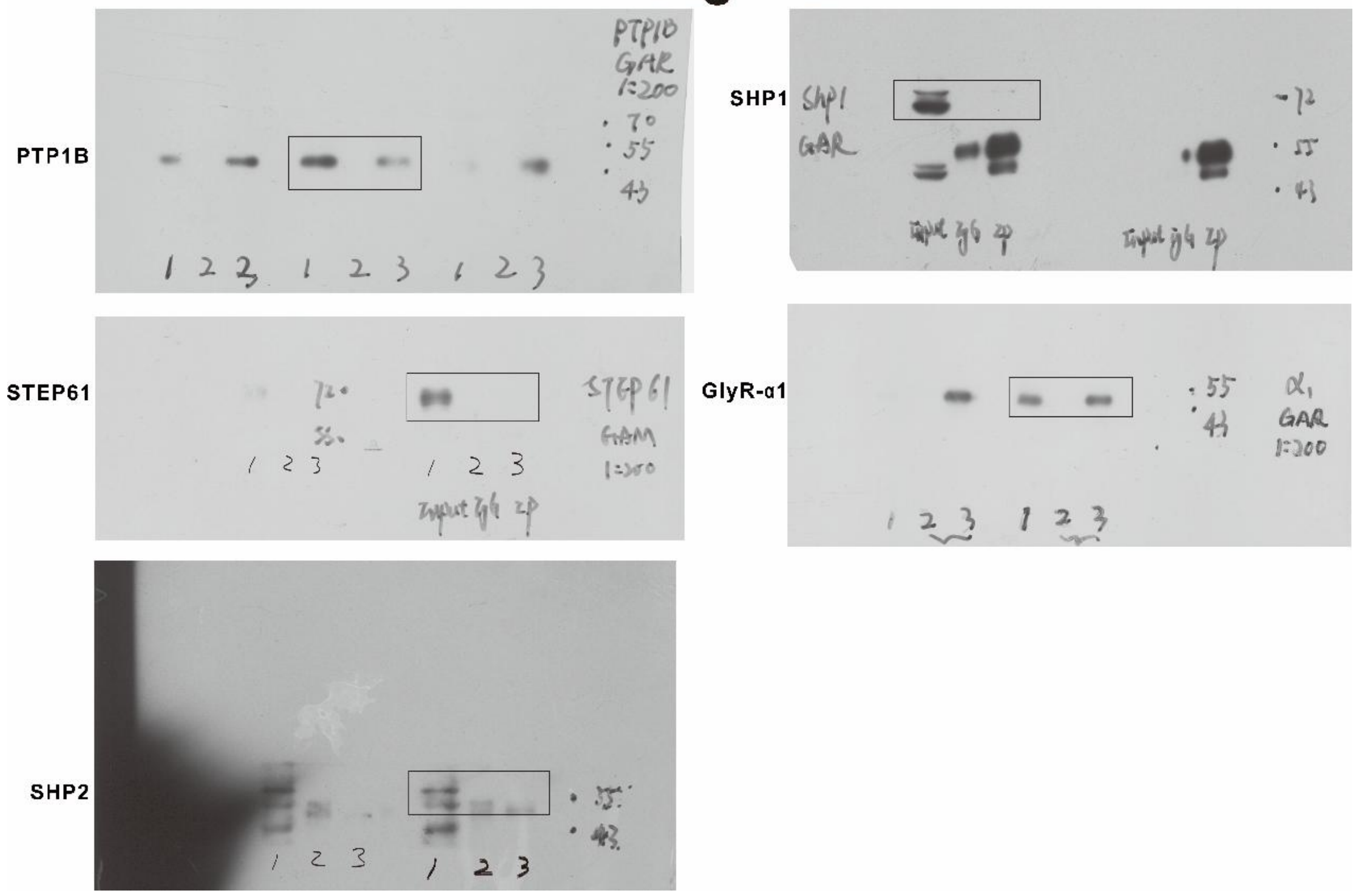

Fig. 1e

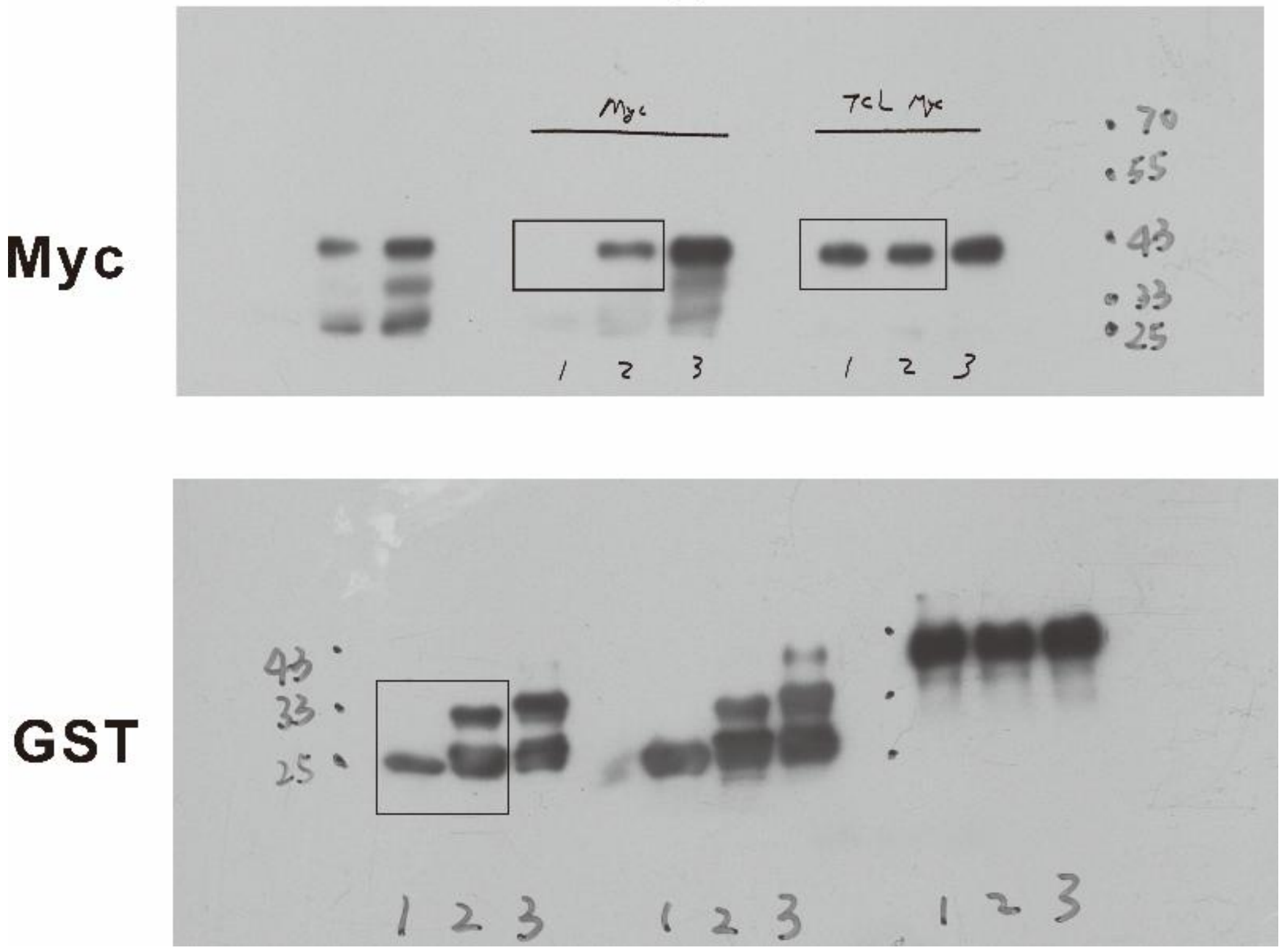

Fig. 2a

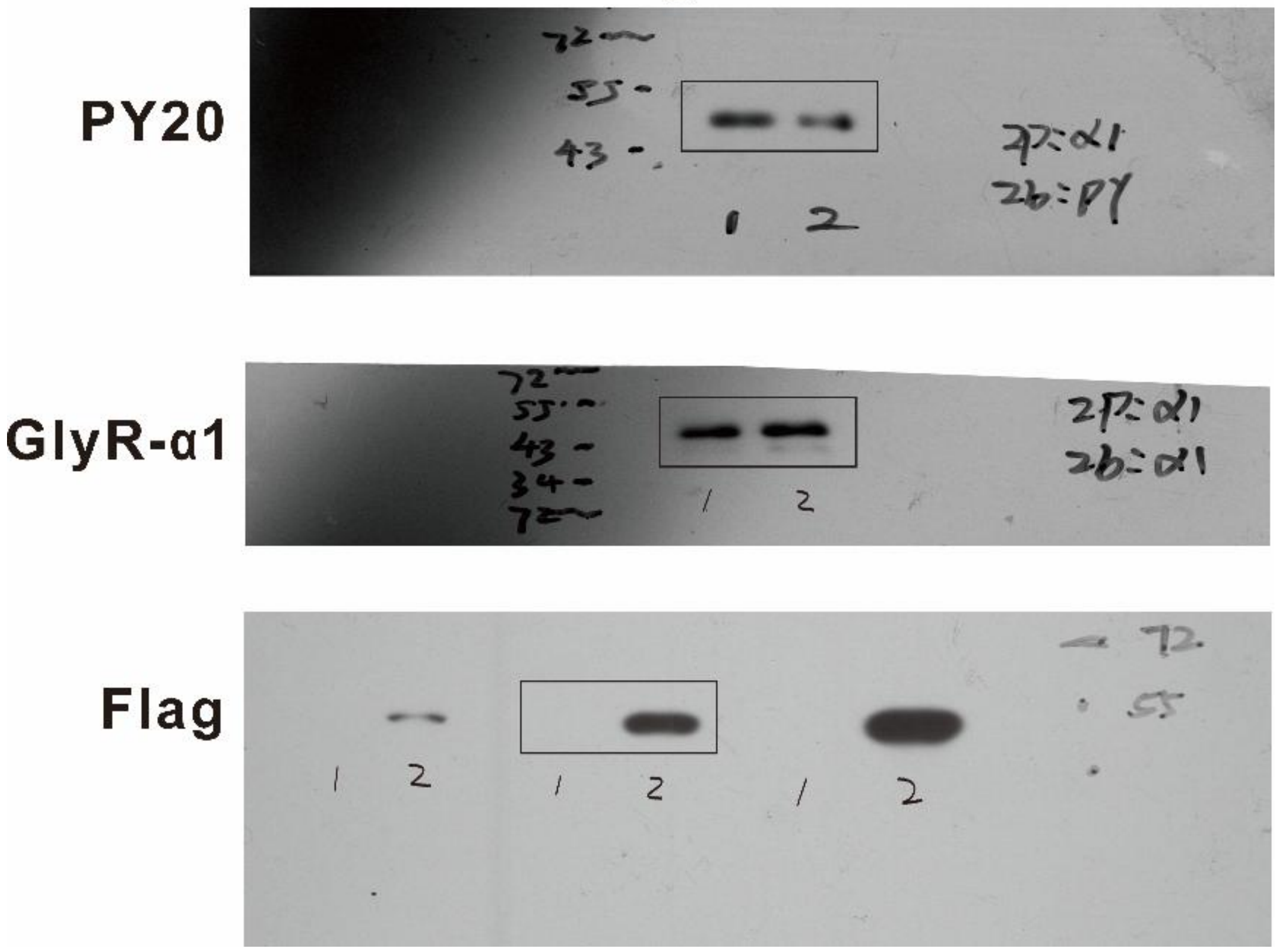

PY20
72
55
43
1
2
GlyR-α1
72
55
43
34
1
2
Flag
72
55
1
2

Fig. 2b

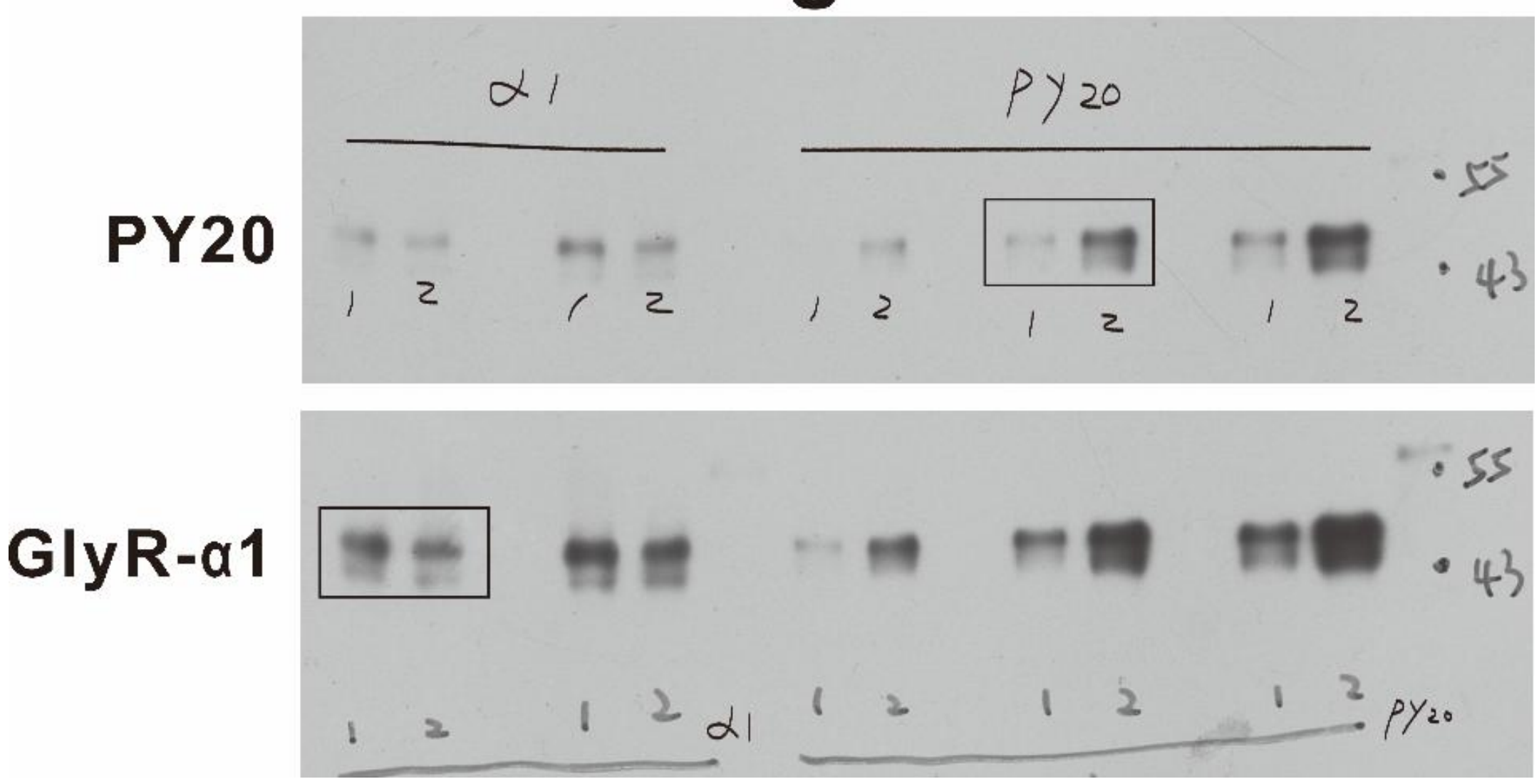

α1
PY20
PY20
55
43
1
2
GlyR-α1
55
43
1
2
α1
PY20

Fig. 3b

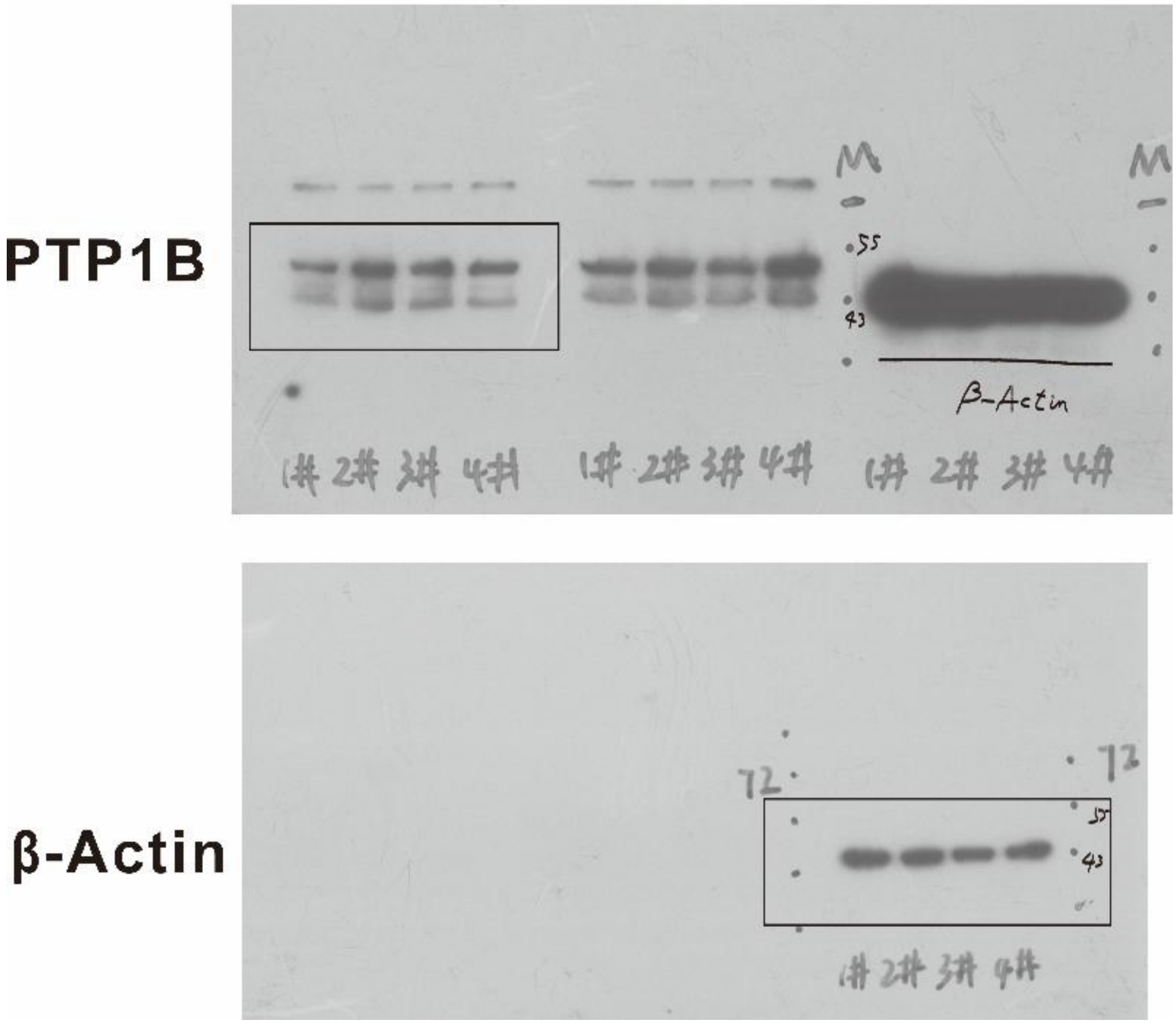
PTP1B
β-Actin
M
55
43
β-Actin
1# 2# 3# 4#
1# 2# 3# 4#
1# 2# 3# 4#
72
72
55
43
1# 2# 3# 4#

Fig. 5b

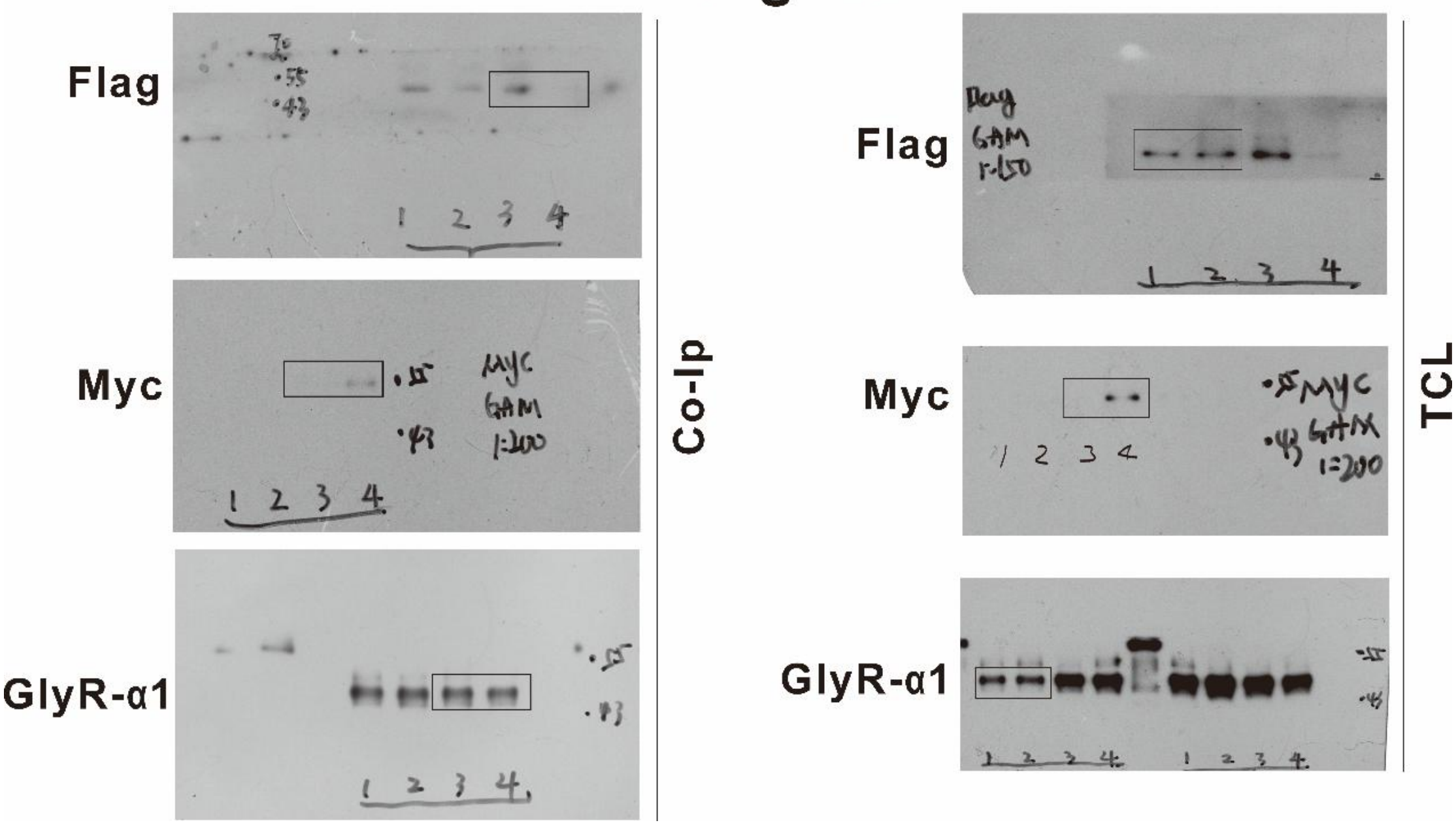
Flag
Myc
GlyR-α1
Co-Ip
TCL
55
43
1 2 3 4
Myc GAM 1:200
1 2 3 4
1 2 3 4
Flag GAM 1:150
1 2 3 4
Myc GAM 1:200
1 2 3 4
1 2 3 4 1 2 3 4

Fig. 5c

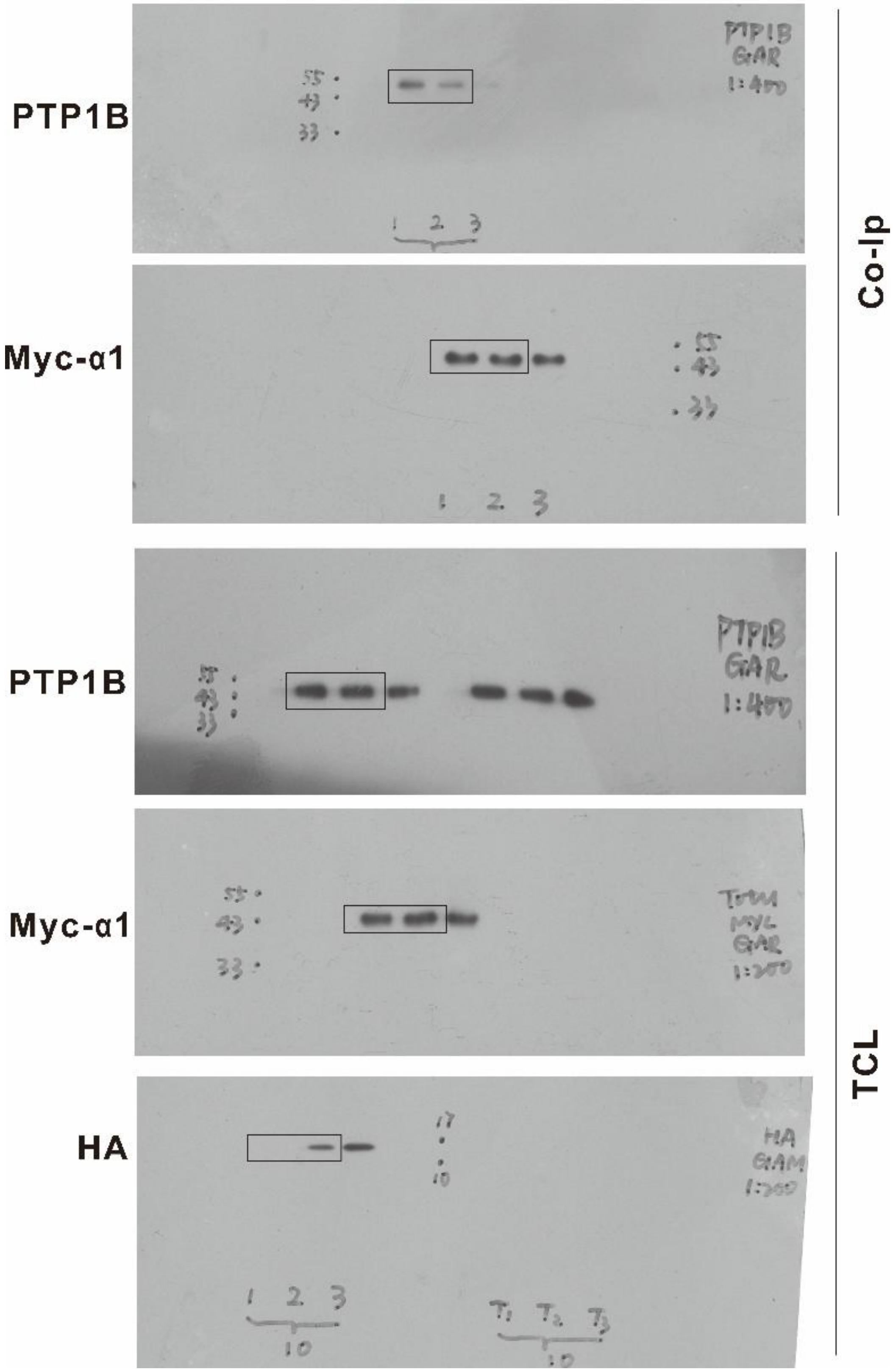

Fig. 5d

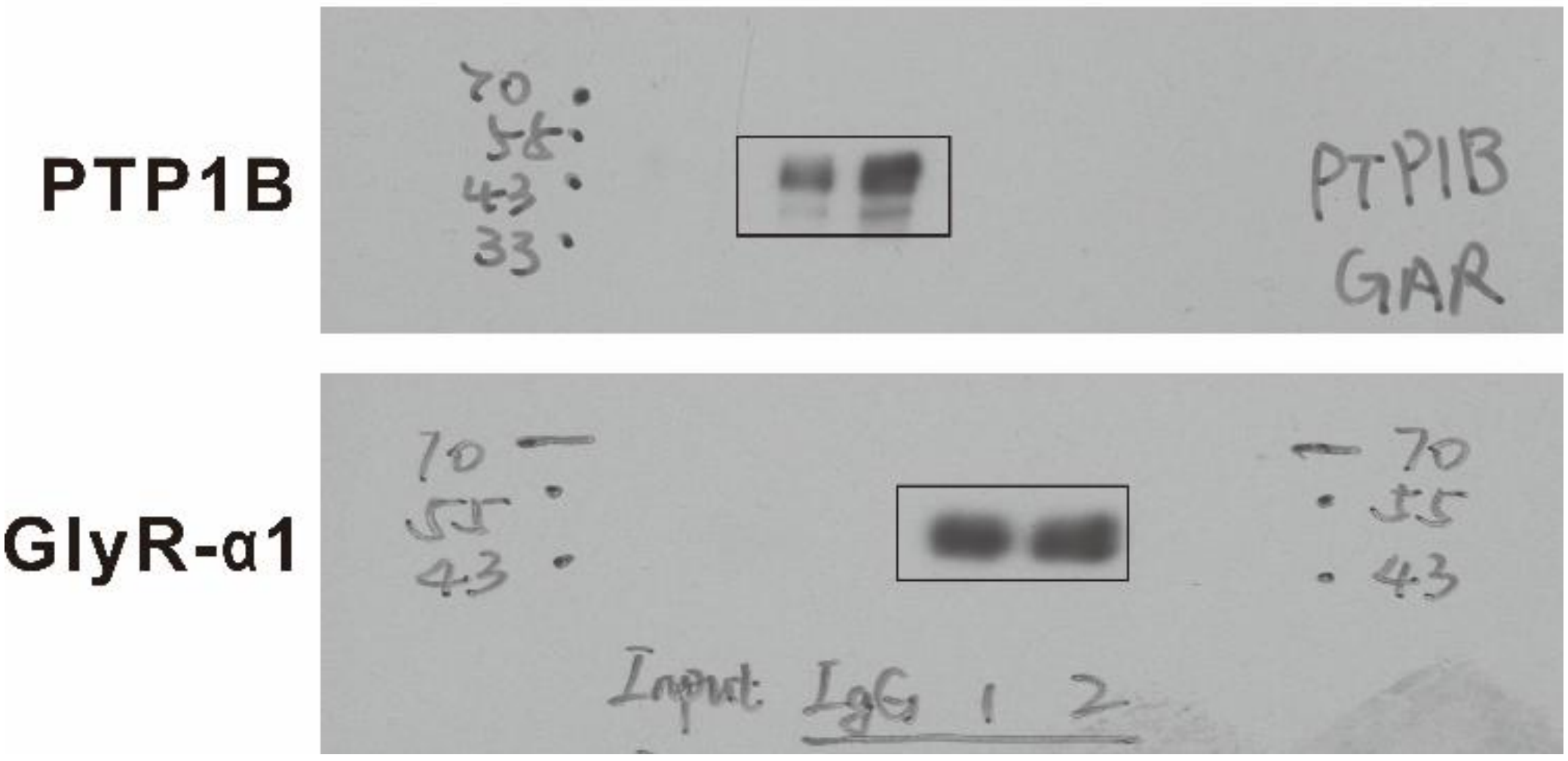

Fig. 6a

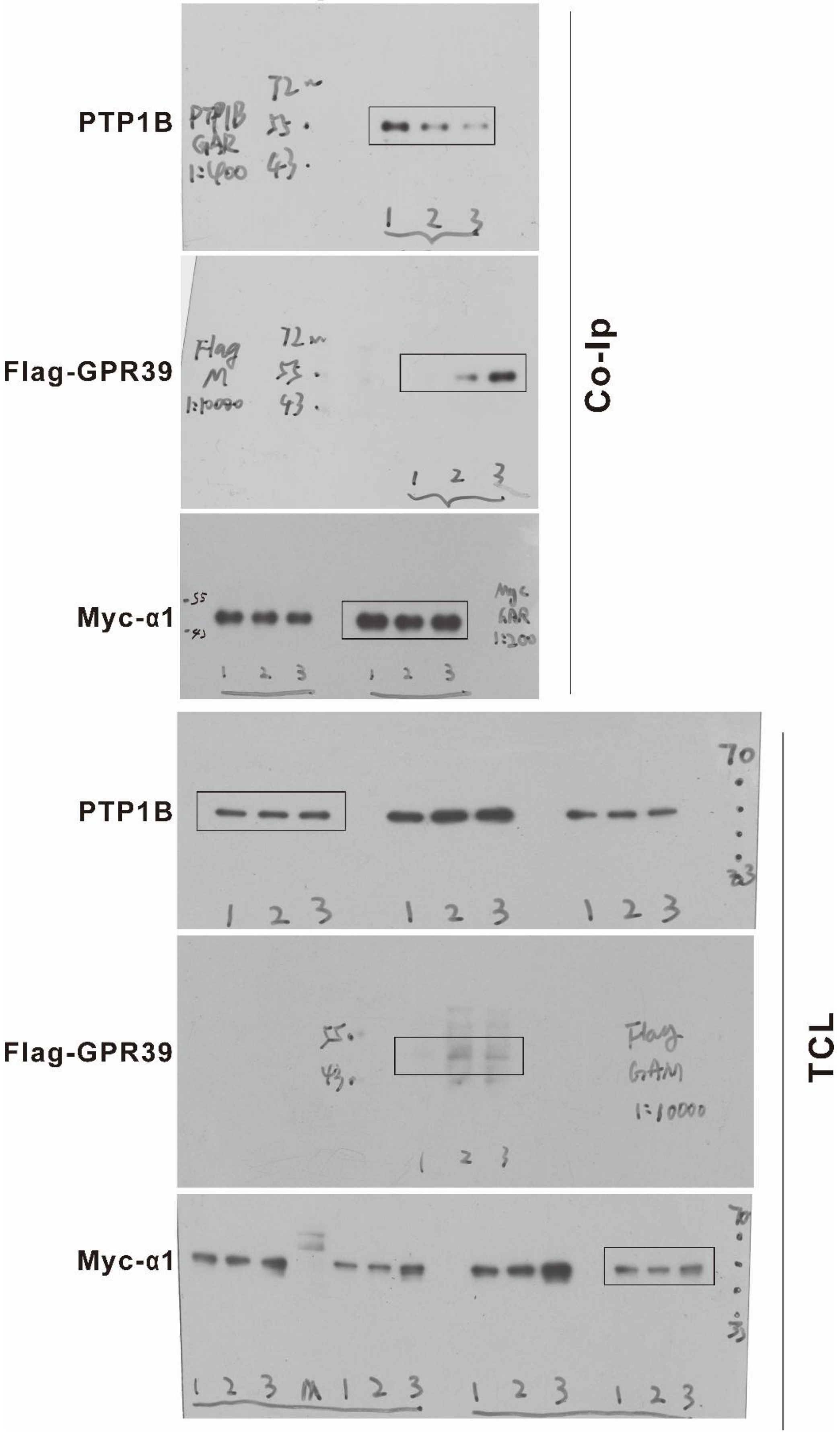

Fig. 6b

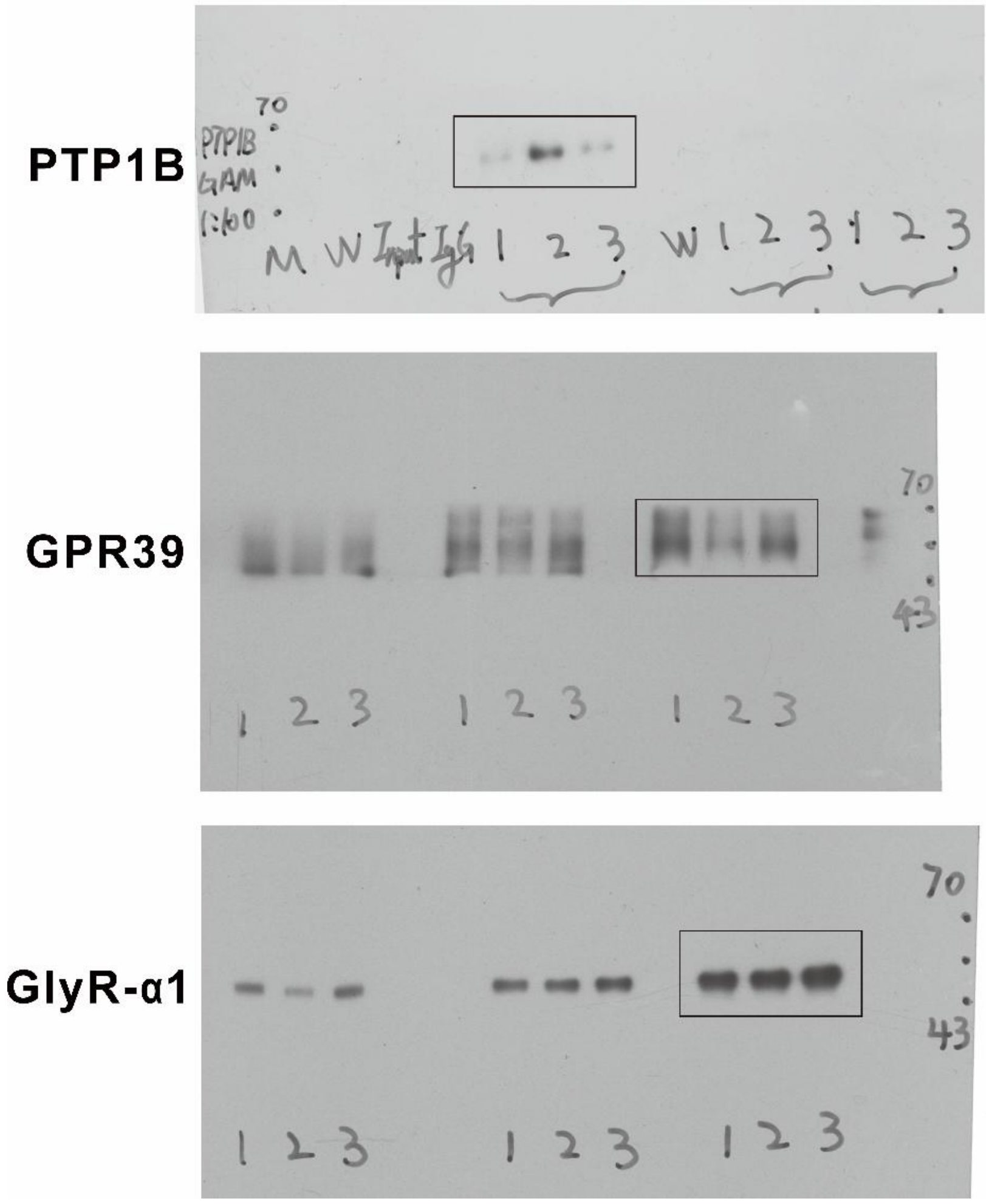
PTP1B
GPR39
GlyR-α1
70
43
PTP1B GAM 1:100
M W Input IgG 1 2 3 W 1 2 3 1 2 3
1 2 3 1 2 3 1 2 3

83
