# Supplementary material for: Enhanced expression of spinal PTP1B contributes to peripheral inflammation- induced glycinergic disinhibition and mechanical pain
Source: Commun Biol. 2026 May 20;9:1080. doi: 10.1038/s42003-026-10318-5 (PMC13458505; doi:10.1038/s42003-026-10318-5)
Supplement: Supplementary file 2 [file 42003_2026_10318_MOESM2_ESM.pdf]

Description of Additional Supplementary Files

File name: numerical source data

Description: The source data behind the graphs in the paper.

File name: Supplementary information

Description: Supplementary figures and legends
