# Supplementary material for: Enhanced expression of spinal PTP1B contributes to peripheral inflammation- induced glycinergic disinhibition and mechanical pain
Source: Commun Biol. 2026 May 20;9:1080. doi: 10.1038/s42003-026-10318-5 (PMC13458505; doi:10.1038/s42003-026-10318-5)
Supplement: Supplementary file 3 [file 42003_2026_10318_MOESM4_ESM.pdf]

nature portfolio

Corresponding author(s): Zhan-Wei Suo

Last updated by author(s): Mar 27, 2026

Reporting Summary

Nature Portfolio wishes to improve the reproducibility of the work that we publish. This form provides structure for consistency and transparency in reporting. For further information on Nature Portfolio policies, see our Editorial Policies and the Editorial Policy Checklist.

Statistics

For all statistical analyses, confirm that the following items are present in the figure legend, table legend, main text, or Methods section.

n/a	Confirmed	
☐	☒	The exact sample size (n) for each experimental group/condition, given as a discrete number and unit of measurement
☐	☒	A statement on whether measurements were taken from distinct samples or whether the same sample was measured repeatedly
☐	☒	The statistical test(s) used AND whether they are one- or two-sided<br>*Only common tests should be described solely by name; describe more complex techniques in the Methods section.*
☒	☐	A description of all covariates tested
☐	☒	A description of any assumptions or corrections, such as tests of normality and adjustment for multiple comparisons
☐	☒	A full description of the statistical parameters including central tendency (e.g. means) or other basic estimates (e.g. regression coefficient) AND variation (e.g. standard deviation) or associated estimates of uncertainty (e.g. confidence intervals)
☐	☒	For null hypothesis testing, the test statistic (e.g. F, t, r) with confidence intervals, effect sizes, degrees of freedom and P value noted<br>*Give P values as exact values whenever suitable.*
☒	☐	For Bayesian analysis, information on the choice of priors and Markov chain Monte Carlo settings
☒	☐	For hierarchical and complex designs, identification of the appropriate level for tests and full reporting of outcomes
☒	☐	Estimates of effect sizes (e.g. Cohen's d, Pearson's r), indicating how they were calculated

Our web collection on statistics for biologists contains articles on many of the points above.

Software and code

Policy information about availability of computer code

Data collection	SuperMaze was used for the analysis of animal behavioral data. LAS X and Image J pro plus were employed for image processing. Image J was used for western blot data. Clampfit 8.0 was used for the acquisition and analysis of electrophysiological data.
Data analysis	SPSS 23.0 was used to analysis data.

For manuscripts utilizing custom algorithms or software that are central to the research but not yet described in published literature, software must be made available to editors and reviewers. We strongly encourage code deposition in a community repository (e.g. GitHub). See the Nature Portfolio guidelines for submitting code & software for further information.

Data

Policy information about availability of data

All manuscripts must include a data availability statement. This statement should provide the following information, where applicable:

- Accession codes, unique identifiers, or web links for publicly available datasets
- A description of any restrictions on data availability
- For clinical datasets or third party data, please ensure that the statement adheres to our policy

All data needed to evaluate the conclusions in the paper are present in the paper.

Research involving human participants, their data, or biological material

Policy information about studies with human participants or human data. See also policy information about sex, gender (identity/presentation), and sexual orientation and race, ethnicity and racism.

Reporting on sex and gender	n/a
Reporting on race, ethnicity, or other socially relevant groupings	n/a
Population characteristics	n/a
Recruitment	n/a
Ethics oversight	n/a

Note that full information on the approval of the study protocol must also be provided in the manuscript.

Field-specific reporting

Please select the one below that is the best fit for your research. If you are not sure, read the appropriate sections before making your selection.

☒ Life sciences ☐ Behavioural & social sciences ☐ Ecological, evolutionary & environmental sciences

For a reference copy of the document with all sections, see nature.com/documents/nr-reporting-summary-flat.pdf

Life sciences study design

All studies must disclose on these points even when the disclosure is negative.

Sample size	The sample size was chosen based on prior similar published studies.
Data exclusions	No data were excluded from the analyses.
Replication	The results in this work are reproducible and have been independently verified by colleagues.
Randomization	The all mice were randomly assigned to experimental groups.
Blinding	The all experiments were conducted blindly by the investigators without knowledge of the manipulations that mice had received.

Reporting for specific materials, systems and methods

We require information from authors about some types of materials, experimental systems and methods used in many studies. Here, indicate whether each material, system or method listed is relevant to your study. If you are not sure if a list item applies to your research, read the appropriate section before selecting a response.

Materials & experimental systems

n/a	Involved in the study	
☐	☒	Antibodies
☐	☒	Eukaryotic cell lines
☒	☐	Palaeontology and archaeology
☐	☒	Animals and other organisms
☒	☐	Clinical data
☒	☐	Dual use research of concern
☒	☐	Plants

Methods

n/a	Involved in the study	
☒	☐	ChIP-seq
☒	☐	Flow cytometry
☒	☐	MRI-based neuroimaging

Antibodies

Antibodies used	rabbit PTP1B antibody (1:400; #B0809, Calbiochem),<br>mouse anti-gephyrin antibody (1:200; Synaptic System; #147021),<br>rabbit anti-GPR39 (1:500; #NLS142; Novus Biologicals),<br>mouse anti-GlyR α1 antibody (1:200; #146111; Synaptic System),

rabbit anti-GlyR α1 (1:200; #146003; Synaptic System),
mouse anti-phosphotyrosine (PY) antibody (1:200; #P4110; Sigma-Aldrich),
mouse anti-PTP1B antibody (1:200; #PH01; Calbiochem),
mouse anti-GST antibody (1:20,000; #E0019; Anbo),
mouse anti-Flag antibody (1:5000; #E0017; Anbo),
mouse anti-Myc antibody (1:600; #sc-40; Santa Cruz),
rabbit anti-Myc antibody (1:1000; #ab9106; Abcam),
mouse anti-HA antibody (1:1000; #66006-1-Ig; Proteintech),
mouse anti-β-actin antibody (1:800; #A5316; Sigma-Aldrich)
rabbit anti-GPR39 antibody (1:200; #NLS142; Novus).
Alexa 488-, Cy3- or Alexa 647-conjugated secondary antibodies (1:500; Invitrogen).~~~

Validation: No validation for commercial antibodies.

Eukaryotic cell lines

Policy information about cell lines and Sex and Gender in Research

Cell line source(s): The HEK293T cells was purchases from the American Type Culture Collection(#CRL-3216)

Authentication: The HEK293T cell lines was used for experiments immediately after obtain.

Mycoplasma contamination: The HEK293T cell lines was used for experiments immediately after obtain.
Therefore, the mycoplasma contamination test is not performed.

Commonly misidentified lines (See ICLAC register): No commonly misidentified cell lines were used.

Animals and other research organisms

Policy information about studies involving animals; ARRIVE guidelines recommended for reporting animal research, and Sex and Gender in Research

Laboratory animals: C57BL/6J mice; SOM-Cre mice; AI14 mice; PTP1B knocknout mice; GPR39 knockout mice; 6-8weeks

Wild animals: No wild animals were used in this study.

Reporting on sex: Male mice were used.

Field-collected samples: The study does not involve samples collected from the field.

Ethics oversight: The animal experiments were approved by the Institutional Animal Care and Use Committee of Lanzhou University.

Note that full information on the approval of the study protocol must also be provided in the manuscript.

Plants

Seed stocks: n/a

Novel plant genotypes: n/a

Authentication: n/a
